# Thermodynamic Analysis of the Adsorption and Micellization Activity of the Mixtures of Rhamnolipid and Surfactin with Triton X-165

**DOI:** 10.3390/molecules27113600

**Published:** 2022-06-03

**Authors:** Edyta Rekiel, Anna Zdziennicka, Katarzyna Szymczyk, Bronisław Jańczuk

**Affiliations:** Department of Interfacial Phenomena, Faculty of Chemistry, Institute of Chemical Sciences, Maria Curie-Skłodowska University in Lublin, 20-031 Lublin, Poland; edyta.rekiel@poczta.umcs.lublin.pl (E.R.); katarzyna.szymczyk@mail.umcs.pl (K.S.); bronislaw.janczuk@poczta.umcs.lublin.pl (B.J.)

**Keywords:** biosurfactants, rhamnolipid, surfactin, adsorption, micellization, standard Gibbs free energy, standard Gibbs free energy of micellization

## Abstract

The surface tension of aqueous solutions of Triton X-165 with rhamnolipid or surfactin mixtures was measured. The obtained results were applied for the determination of the concentration and composition of the Triton X-165 and biosurfactants mixture at the water–air interface as well as the contribution of the particular component of the mixtures to water surface tension reduction and the mutual influence of these components on the critical micelle concentration. The determination of these quantities was based on both the commonly used concepts and a new one proposed by us, which assumes that the composition of the mixed monolayer at the water–air interface depends directly on the pressure of the monolayer of the single mixture component and allows us to determine the surface concentration of each mixture component independently of surface tension isotherms shape. Taking into account the composition of the mixed monolayer at the water–air interface, the standard Gibbs adsorption free energy was considered. The obtained results allow us to state that the concentration of both mixture components corresponding to their saturated monolayer and the surface tension of their aqueous solution can be predicted using the surfactants’ single monolayer pressure and their mole fraction in the mixed monolayer determined in the proposed way.

## 1. Introduction

Biosurfactants are characterized by very good interfacial and aggregation properties as well as their biodegradability [1,2,3,4,5]. This promotes their application, among other things, in crude oil recovery [6,7], in the pharmaceutical industry [8], or in natural environment bioremediation [9]. Moreover, they are found in medical [10,11,12] and household products [10]. In numerous practical applications, the surface tension of the aqueous solution of biosurfactants or their mixtures with classical synthetic surfactants plays a very important role. The proper surface tension value of the aqueous solution required for a given practical application can be obtained at a considerably smaller concentration of biosurfactants than in the case of synthetic surfactants. However, due to the biosurfactants’ production costs, their individual practical applications are confined. Therefore, the mixtures of biosurfactants with classical synthetic surfactants are more and more often used in practice [13,14,15,16]. From practical and theoretical points of view, it is important to establish the composition and concentration of a biosurfactant and a classical surfactant in the aqueous solution required to achieve the proper surface tension values of this solution on the basis of the surface tension isotherm of the aqueous solution of the mixture components. In practice, it is particularly essential to determine the value of the surface tension of the solution at which the process of the surfactants’ aggregation takes place. This problem can be solved to a much greater extent in the case of the mixture of synthetic surfactants than those of biosurfactants and classical surfactants. To predict the isotherms of the surface tension of the aqueous solution of the biosurfactants and classical surfactant mixture, and thus the required value of the solution surface tension, the relationship between the surface tension isotherm of the aqueous solution of particular components of the mixture and the mixture itself should be known. To study this issue, rhamnolipid (RL), surfactin (SF), and Triton X-165 (TX165) were chosen.

The RL and SF are the most prominent representatives of biosurfactants. Rhamnolipid is produced by *Pseudomonas aeruginosa* [17,18,19]. In turn, surfactin is produced mainly by *Bacillus subtilis* [8,20,21]. RL reduces the water surface tension due to its adsorption at the water–air interface to a minimal value, even lower than that obtained for the nonionic synthetic surfactants such as Triton [22,23]. The maximal reduction in the water surface tension by SF adsorption at the water–air interface is similar to that of Triton X-100 [22,23]. The critical micelle concentration of RL and SF (CMC) is about two orders of magnitude smaller than that of classical surfactants [22,24]. Biosurfactants can positively affect the adsorption and aggregation properties of classic surfactants. For this reason, the literature reports some studies on the adsorption and aggregation behavior of biosurfactant mixtures with different kinds of synthetic surfactants [16,25,26]. Mostly, they deal with the possible occurrence of synergy in the water surface tension reduction and in the aggregation process. As for the synergistic effects, some surface tension isotherms of the biosurfactant and classic surfactant mixtures, in the concentration range of mixtures of a given composition up to CMC with the linear dependence between the surface tension ($\gamma_{LV}$) and the logarithm of the concentration (*C*), are taken into account [27]. On the other hand, it is difficult to find in the literature isotherms of $\gamma_{LV}$ of the aqueous solutions of biosurfactant and classic surfactant mixtures in the concentration range at which both the unsaturated and saturated mixed monolayers are formed at the water–air interface. Moreover, the literature lacks a description and/or prediction of the isotherms of the surface tension of the biosurfactants and classical surfactant mixtures as well as the data about the composition of the mixed monolayer at the water–air interface. It should also be mentioned that the analysis of the surface tension of the isotherms of the surfactant mixture based on the isotherm of its particular components only at the constant composition of the mixture, which is mainly investigated, provides a complete explanation of the mixture surface behavior. Therefore, the aim of the study was to measure $\gamma_{LV}$ of the aqueous solution of the biosurfactant and nonionic classical surfactant mixtures, both at the changing concentration of the mixtures at their constant composition and the constant concentration of one component of the mixture and the variable of the other one. The measurements of $\gamma_{LV}$ were made in a wide range of composition and concentration. Moreover, the obtained $\gamma_{LV}$ isotherms were analyzed regarding their possible description and/or prediction to determine the composition of the mixed monolayer at the water–air interface and to find a possible existence of the synergetic effect in the water surface tension and in the CMC reduction. Thus, the mixtures of RL with TX165 and SF with TX165 were applied. The chosen biosurfactants are anionic, including, among others, the –COOH group in their molecules. However, the TX165 molecules contain oxyethylene groups. Under some conditions, the hydrogen ions can be joined with the oxyethylene group and TX165 can behave as the cationic surfactant [27]. Thus, the attractive electrostatic interactions between the RL or SF and TX165 molecules, apart from the formation of hydrogen bonds between them, are possible.

## 2. Results and Discussion

### 2.1. Comparison of Some Physicochemical Properties of Solution Components

**Figure 1:**
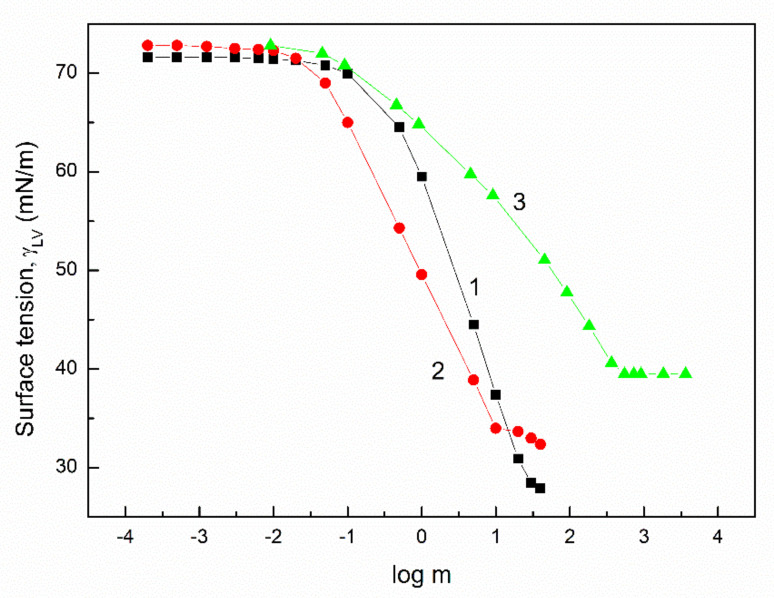
A plot of the surface tension ($\gamma_{LV}$) of the aqueous solution of RL (curve 1), SF (curve 2), and TX165 (curve 3) vs. the logarithm from the surfactant weight in mg/dm^3^ (m).

Surfactants and biosurfactants differ from other substances because of their tendency toward adsorption at different interfaces and their ability to aggregate in a largely polar liquid environment such as water. The adsorption and aggregation properties of surfactants and biosurfactants depend on the type and amount of various chemical groups present in the hydrophobic and hydrophilic parts of their molecules, the size of the molecules, and the presence of an electric charge, as well as the parameters and components of the surface tension. Similar to RL and SF, chosen by us for studies on the adsorption and aggregation properties of their mixtures with the nonionic TX165 surfactant, the ionic biosurfactants have much better adsorption and aggregation properties than the synthetic surfactants. They reduce the water surface tension to a given value at a concentration considerably smaller than that of TX165 (Figure 1) [22,23]. For example, the reduction in water surface tension to a value equal to 55 mN/m takes place at a TX165 concentration 8.5 times greater than that of RL and 69.8 greater than that of SF (Figure 1). In the case of the critical micelle concentration (CMC) (Table 1) [22,24], the ratio of the TX165 CMC value to RL and SF as well as the CMC of RL to SF is equal to 10.4, 56, and 5.4, respectively.

What can be the reason for such a large difference between the TX165 and biosurfactants concentration needed to reduce the water surface tension to a given value and between the CMC values? The TX165, RL, and SF tendency to adsorb at the water–air interface is similar because the standard Gibbs free energy of their adsorption calculated from the Langmuir equation modified by de Boer is comparable (Table 1) [22,23]. This indicates that the transition of one TX165, RL, and SF molecule from the bulk phase of the solution to the water–air interface causes similar changes in the Gibbs free energy of the solution. These changes result from the hydration degree of hydrophobic and hydrophilic parts of the biosurfactants and surfactant molecules.

The number of water molecules in direct contact can be approximately established based on the water, biosurfactant, and surfactant contactable area. The minimal contactable area of the water molecule at 293 K is equal to 10 Å^2^. The contactable area of the TX165, RL, and SF molecules can be approximately established based on the length of the chemical bonds between the individual atoms in the molecule, the angle between these bonds, as well as the average distance between the biosurfactant, surfactant, and other molecules. It appears that the volume of the surfactant molecule in the aqueous environment can be determined based on the cube in which the surfactant molecule is inscribed, or the sum of the cubes in which the individual parts of the surfactant are inscribed. The volumes of the TX165, RL, and SF moles determined in this way are close to their partial molar volume [24,28] (Table 1). Thus, it was possible to establish the contactable area of TX165, RL, and SF molecules (Table 1). Taking into account the contactable area of the hydrophobic part of these compounds and water, it can be stated that about 36, 30, and 34 water molecules can be contacted directly with the hydrophobic parts of the TX165, RL, and SF molecules, respectively. As the hydration of the hydrophobic parts of the surfactants exerts the main influence on the standard Gibbs free energy of adsorption, their values for the studied compounds are close. This fact does not account for the difference in the water surface tension reduction by TX165, RL, and SF adsorption at the water–air interface. It is commonly known that the water surface tension results from the Lifshitz–van der Waals and acid–base intermolecular interactions.

The acid–base intermolecular interactions are associated with hydrogen bonds. According to the van Oss and Constanzo concept [29], the surface tension of biosurfactants and surfactants depends on the orientation of their molecules towards the air phase. This leads to the concept of the head and tail of surfactant surface tension. The surface tension of the tail of TX165, RL, and SF results from the Lifshitz–van der Waals intermolecular interactions and its value is smaller than that of the Lifshitz–van der Waals component of the water surface tension (Table 1) [30,31,32]. However, the differences between the values of the Lifshitz–van der Waals component for TX165, RL, and SF surface tension are not great for justifying the differences in the water surface tension reduction (Table 1, Figure 1). The difference is found particularly in the concentration range corresponding to that of the saturated monolayer at the water–air interface (Table 1). It can be assumed that the molecules of surface-active compounds are oriented perpendicular to the water–air interface, and the hydrophobic parts are in the air phase in the saturated monolayer. In such a case, the limiting area occupied by one TX165 molecule is about two times smaller than that of RL and 3.4 times smaller than that of the SF molecule. One TX165 molecule can replace 3.5 molecules of water, seven for RL, and for SF as many as 12 molecules of water at the water–air interface can be replaced. This indicates that at the same concentration of TX165, RL and SF in the monolayer, the ratio of the water–air interface covered by these compounds increases from TX165 to SF. This fact may be one of the reasons for the increase in the degree of surface tension reduction by adsorbing the TX165, RL and SF molecules in the order from TX165 to SF. However, the maximal Gibbs surface excess concentration decreases in the order from TX165 to SF (Table 1) [22,23]. In fact, the maximum fraction of the interface area occupied by the RL molecules is almost twice as large as that of TX165 but the fraction of the area occupied by the SF molecules is slightly smaller than that of RL (Table 1). Taking into account the Lifshitz–van der Waals component (LW) of the water surface tension and the tail of TX165, RL and SF surface tension, it can be stated that the LW component of the water surface tension can change theoretically as a function of TX165, RL, and SF concentration from 26.85 to 22.00 mN/m, from 26.85 to 21.80 mN/m, and from 26.85 to 24.70 mN/m, respectively (Table 1) [24,25,26]. On the other hand, the acid–base component of the water surface tension can be changed from 45.95 mN/m to zero as a function of their concentrations. It should be mentioned that the LW component of the TX165, RL, and SF head surface tension is greater than for water. The minimal surface tension of the aqueous solution of TX165, RL, and SF is higher than that of their tail (Table 1) [30,31,32]. This indicates that the hydrogen bonds between the water molecules are not completely reduced by the TX165, RL, and SF adsorption at the water–air interface. It seems also that the ability to reduce the acid–base component of the water surface tension is the main reason for the differences in the surface activity of TX165, RL, and SF. A great difference in the kind and amount of the polar and apolar groups in the hydrophilic parts of the TX165, RL, and SF molecules can be observed.

In the case of the aggregation properties of TX165, RL, and SF, the ability to form aggregates in the aqueous solution increases from TX165 to SF. This is in accordance with the changes in the standard Gibbs free energy of their micellization (Table 1) [16,24,25,26,27,28]. 

### 2.2. Surface Tension of TX165 Mixtures with Rhamnolipid and Surfactin

**Figure 2:**
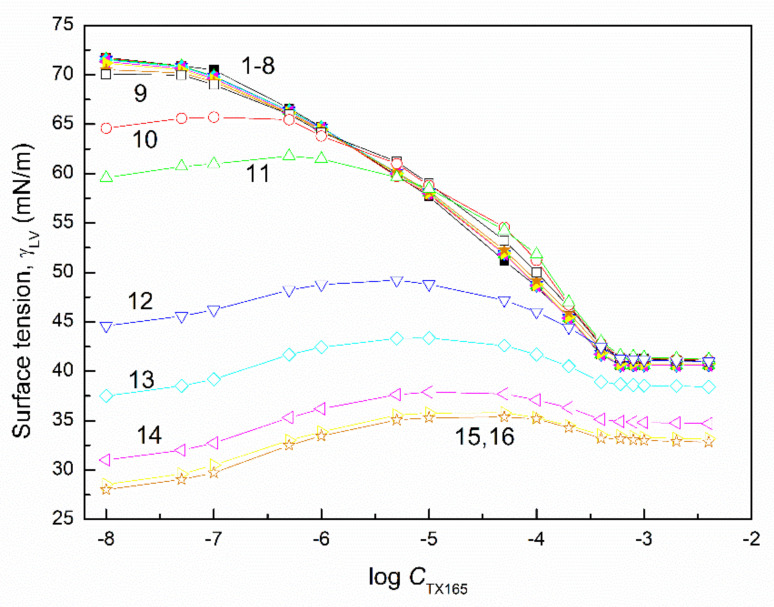
A plot of the surface tension ($\gamma_{LV}$) of the aqueous solution of the RL and TX165 mixture vs. the logarithm of the TX165 concentration ($C_{\mathrm{TX}165}$). Curves 1−16 correspond to the constant RL concentration equal to 0.0002, 0.0005, 0.00125, 0.003, 0.00625, 0.01, 0.02, 0.05, 0.1, 0.5, 1, 5, 10, 20, 30, and 40 mg/dm^3^, respectively.

**Figure 3:**
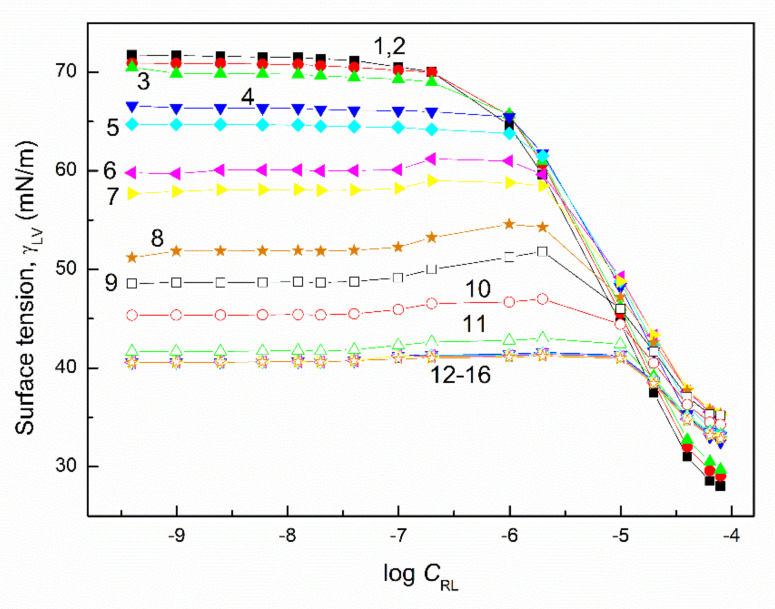
A plot of the surface tension ($\gamma_{LV}$) of the aqueous solution of the RL and TX165 mixture vs. the logarithm of the RL concentration ($C_{\mathrm{RL}}$). Curves 1−16 correspond to the constant TX165 concentration equal to 1 × 10^−8^, 5 × 10^−8^, 1 × 10^−7^, 5 × 10^−7^, 1 × 10^−6^, 5 × 10^−6^, 1 × 10^−5^, 5 × 10^−5^, 1 × 10^−4^, 2 × 10^−4^, 4 × 10^−4^, 6 × 10^−4^, 8 × 10^−4^, 0.001, 0.002, and 0.004 mole/dm^3^, respectively.

**Figure 4:**
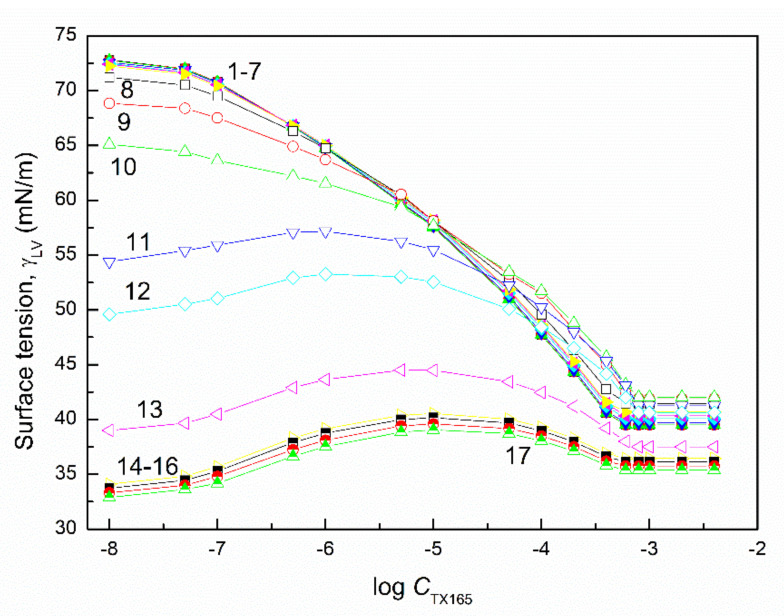
A plot of the surface tension ($\gamma_{LV}$) of the aqueous solution of the SF and TX165 mixture vs. the logarithm of the TX165 concentration ($C_{\mathrm{TX}165}$). Curves 1−16 correspond to the constant SF concentration equal to 0.0002, 0.0005, 0.00125, 0.003, 0.00625, 0.01, 0.02, 0.05, 0.1, 0.5, 1, 5, 10, 20, 30, and 40 mg/dm^3^, respectively.

**Figure 5:**
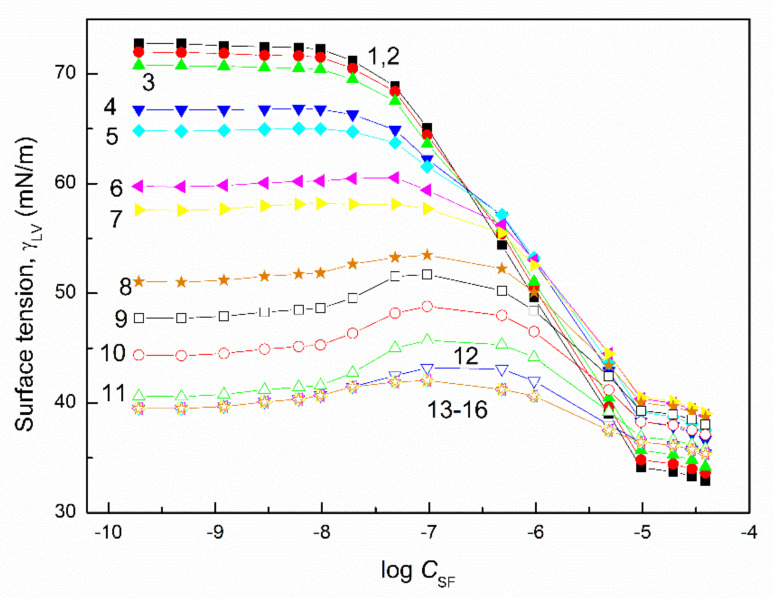
A plot of the surface tension ($\gamma_{LV}$) of the aqueous solution of the SF and TX165 mixture vs. the logarithm of the SF concentration ($C_{\mathrm{SF}}$). Curves 1−16 correspond to the constant TX165 concentration equal to 1 × 10^−8^, 5 × 10^−8^, 1 × 10^−7^, 5 × 10^−7^, 1 × 10^−6^, 5 × 10^−6^, 1 × 10^−5^, 5 × 10^−5^, 1 × 10^−4^, 2 × 10^−4^, 4 × 10^−4^, 6 × 10^−4^, 8 × 10^−4^, 0.001, 0.002, and 0.004 mole/dm^3^, respectively.

**Figure 6:**
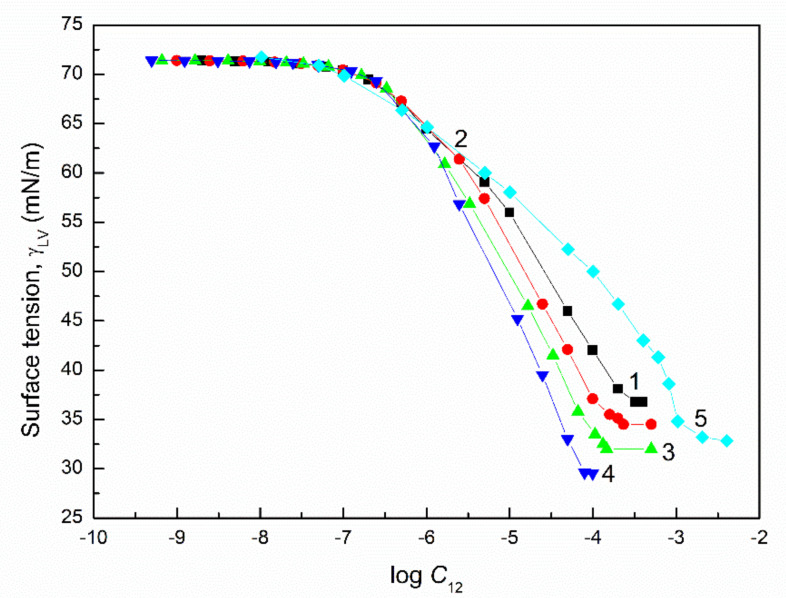
A plot of the surface tension ($\gamma_{LV}$) of the aqueous solution of the RL and TX165 mixture vs. the logarithm of its concentration ($C_{12}$). Curves 1−4 correspond to the RL mole fractions in the mixture equal to 0.2, 0.4, 0.6, and 0.8, respectively. Curve 5 corresponds to the sum of the RL and TX165 concentrations, where the TX165 concentration changed from 0 to 0.004 mole/dm^3^ and the RL concentration changed from 3.97 × 10^−10^ to 7.94 × 10^−5^ mole/dm^3^, as applied in the literature [22,24] for their aqueous solution surface tension measurements.

**Figure 7:**
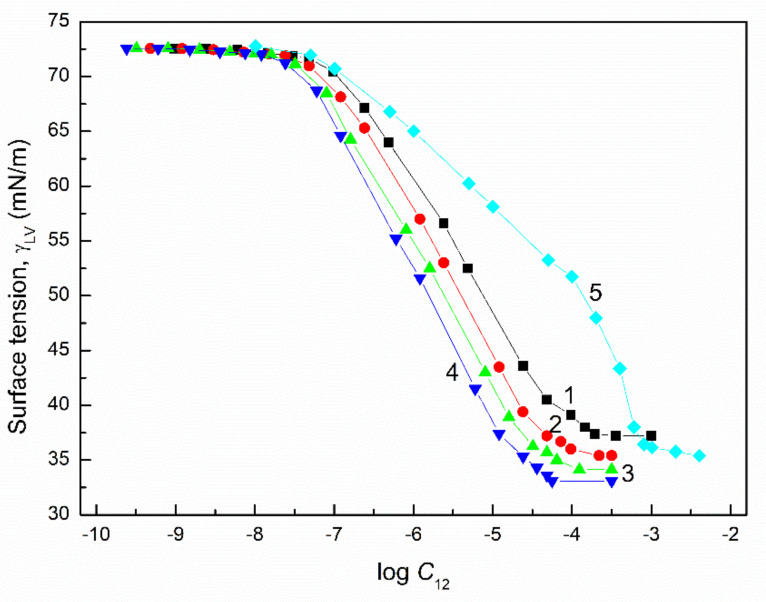
A plot of the surface tension ($\gamma_{LV}$) of the aqueous solution of the SF and TX165 mixture vs. the logarithm of its concentrations ($C_{12}$). Curves 1−4 correspond to the SF mole fractions in the mixture equal to 0.2, 0.4, 0.6, and 0.8, respectively. Curve 5 corresponds to the sum of the SF and TX165 concentrations, where the TX165 concentrations changed from 0 to 0.004 mole/dm^3^ and the SF concentration changed from 1.93 × 10^−10^ to 3.86 × 10^−5^ mole/dm^3^, as applied in the literature [22,24] for their aqueous solution surface tension measurements.

The surface tension of the aqueous solution of the TX165 with RL and TX165 with SF mixtures ($\gamma_{LV}$) was considered at both the constant concentration of biosurfactants, the changing TX165 concentration and vice versa (Figure 2, Figure 3, Figure 4 and Figure 5), and the constant composition of the mixture as a function of its concentration (Figure 6 and Figure 7). However, the concentration of the TX165 + biosurfactant mixtures changed depending on the mixture composition. In other words, the biosurfactant concentration was the same in different compositions of the mixture with TX165. The concentration of TX165 was relative to that of the biosurfactant concentration but different for each mixture composition. The TX165 concentration was selected so as to obtain mixtures with biosurfactant mole fractions equal to 0.2, 0.4, 0.6, and 0.8. Additionally, the surface tension of the TX165 with the biosurfactant mixture, in which the concentration of the particular component was the same as in the solutions of single compounds, was measured [22,24] (Figure 6 and Figure 7). 

The shape of the $\gamma_{LV}$ isotherms of the aqueous solution of the TX165 mixtures with the biosurfactants at their constant concentration in the range from zero to that at which the saturated monolayer of biosurfactants is formed ($C_{min}^{sat}$) (Table 1) [22] in the absence of TX165 is similar to that of the aqueous solution of single TX165. However, above $C_{min}^{sat}$, some maxima on the isotherms of surface tension are observed (Figure 2 and Figure 4). These maxima are more and more visible with the increasing values of the constant biosurfactant concentration. In the case of the surface tension isotherms of the aqueous solution of TX165 mixtures with the biosurfactants at a constant TX165 concentration (Figure 3 and Figure 5), the same relations as at the constant biosurfactant concentration are observed. At a constant TX165 concentration smaller than $C_{min}^{sat}$, the shape of the $\gamma_{LV}$ isotherms are similar to that for the single biosurfactants. Above the $C_{min}^{sat}\;$of TX165, some maxima on the $\gamma_{LV}$ isotherms can be seen. They do not indicate a decrease in the TX165 adsorption from its mixture with biosurfactants in the concentration range from zero to that corresponding to the maximal value of $\gamma_{LV}$. This may result from the great difference as regards the adsorption activity of TX165 and the biosurfactants. 

In the case of the aqueous solutions at the constant composition of TX165 mixtures with biosurfactants, the shape of $\gamma_{LV}$ isotherms are rather similar to those of the aqueous solutions of biosurfactants compared to that of the TX165 solution (Figure 6 and Figure 7). This likely results from the fact that at the comparable concentration of biosurfactants and TX165, as a result of the higher adsorption activity of biosurfactants than that of TX165, there is a greater effect on the shape of the mixture solution isotherms compared to TX165.

It is very important to describe and/or predict the isotherm of $\gamma_{LV}$ for a more detailed consideration of the adsorption behavior and properties of the mixed monolayer at the water–air interface. It appears that the isotherms of the surface tension of the aqueous solution of the TX165 mixture with the biosurfactants can be described by the exponential function of the second order. However, in the case of the isotherms on which the maxima of $\gamma_{LV}$ were observed, it was impossible to describe the isotherms of the surface tension by one exponential function of the second order in the whole mixture concentration range (Figure 2, Figure 3, Figure 4 and Figure 5). In the case of the aqueous solution of TX165 mixtures with RL and/or SF in which the concentration of one mixture component was constant, the $\gamma_{LV}$ isotherms were described by the exponential function of the second order obtained, taking into account both the changing concentration of one component of the mixture and the summed concentration of the two components of the mixture (Figures S1–S12). It appears that the values of the surface tension of the aqueous solution of the TX165 with RL or SF mixtures, determined by the exponential function of the second order, in the case in which only the changing concentration of one component of the mixture is taken into account, are closer to those measured than in the case when the total concentration of the mixture is applied. The description of the $\gamma_{LV}$ isotherms with the maxima by the second-order exponential function is more complicated than in the case of the isotherms without the maxima. These isotherms can be described only by two different second-order exponential functions (Figures S2, S3, S5, S6, S8, S9, S11, S12). 

The equation of the exponential function of the second order which includes $y_{0}$, $A_{1}$, $A_{2}$, $t_{1}$, and $t_{2}$ constants has the form:

(1) $$\gamma_{LV}=y_{0}+A_{1}\exp\left(\frac{-C}{t_{1}}\right)+A_{2}\exp\left(\frac{-C}{t_{2}}\right)\;$$

where *C* is the concentration of the surfactant or mixture of surfactants.

It was stated that the standard Gibbs free energy of surfactants depends on the surface tension of tails and tail–water interface tension [30,31,32]. Therefore, it seems that the constants in Equation (1) are associated with the components and parameters of water as well as the tail and head of the surfactants’ surface tension. The analysis of the constants in Equation (1) for the aqueous solutions of TX165 and biosurfactant mixture in which the concentration of one component of the mixture is constant and that of the other one is variable is difficult. For these solutions, the concentration and composition of the mixture change. Therefore, the constants in Equation (1) are considered only for the aqueous solution of TX165 with the biosurfactant mixtures at the constant composition and variable concentration (Figures S13–S17). For both the TX165 with RL and TX165 with SF mixtures, the constant $y_{0}$ decreases as a function of the mole fraction of biosurfactant in the mixture in the bulk phase. In the case of the TX165 + SF mixture, the relationship between $y_{0}$ and the mole fraction of the biosurfactant is almost linear (Figure S13). It seems that this constant is related to the Lifshitz–van der Waals component of the tail of the surfactant’s surface tension and water–tail interface tension. The Lifshitz–van der Waals interactions are directly associated with the minimal surface tension value of the surfactants and their mixture’s aqueous solution. The $y_{0}$ values are close to those of the minimal surface tension of the aqueous solution of the TX165 + RL and TX165 + SF mixtures (Figure 6, Figure 7, and Figure S13). The other constants in Equation (1) may result from the acid–base components of the surfactants’ head surface tension and the electrostatic interactions. The changes of $A_{1}$, $A_{2}$, $t_{1}$, and $t_{2}$ as a function of the composition of the TX165 + RL and TX165 + SF mixtures are not linear. Some maxima and minima are observed (Figures S14–S17). However, so far, it has been difficult to express the constants in Equation (1) as a function of some properties of surfactants and biosurfactants.

The isotherms of the $\gamma_{LV}$ of the aqueous solution of many surfactants are often described by the Szyszkowski equation [27]. However, in the case of the aqueous solution of surfactant mixtures, fewer attempts to describe the $\gamma_{LV}$ of these solutions by the Szyszkowski equation are reported in the literature [33]. It is commonly known that $\gamma_{LV}$ in the Szyszkowski equation depends on the maximal Gibbs surface excess concentration ($\Gamma^{max}$), the concentration of surfactants in the bulk phase (C), and the standard Gibbs free energy of adsorption ($\Delta G_{ads}^{0}$), which is represented by the constant a in this equation. The Szyszkowski equation can be expressed as [27]:

(2) $$\gamma_{0}-\gamma_{LV}=RT\ln\Gamma^{max}\ln\left(\frac{C}{a}+1\right)$$

where $\gamma_{0}$ is the solvent surface tension and n is the parameter used in the Gibbs isotherm equation for the determination of the surface excess concentration of the given surfactants and the mixture of surfactants.

The value of n for the chosen biosurfactants is equal to 2 because they are the 1: 1 type of electrolyte. For TX165 with biosurfactant mixtures, n changes from 1 to 2 as a function of the mixture’s composition. The use of the Szyszkowski equation for the calculation of the surface tension of the aqueous solutions of mixtures of non-ionic surfactants with the biosurfactants examined by us is not easy. Firstly, in the case of a series of the aqueous solutions of TX165 mixtures with biosurfactants, in which the concentration of one of the components is constant and the other changes, it is difficult to determine the concentration range of the mixture in which its components are in the monomeric form in the bulk phase. The surface-active substance only in the monomeric form influences the amount of adsorption, which is connected with the water surface tension reduction [27]. This fact is not often taken into account. Thus, it is not possible to describe the surface tension isotherm by the Szyszkowski equation in the whole concentration range of a given surfactant. Secondly, the problem is to establish the $\gamma_{0}$ value for a series of aqueous solutions of TX165 mixtures with RL or SF if the concentration of one component is constant and that of the other one changes. There are two possibilities. One is to take $\gamma_{0}$ as the surface tension of the water and the other is to use $\gamma_{0}$ as the value of the surface tension of the aqueous solution of the single component at its concentration being constant in the aqueous solution of the binary mixture. When solving the Szyszkowski equation in relation to the surface tension of the binary mixture solution at the constant concentration of one of the components, the third problem is what concentration should be used in this equation for calculations—the total or only that of the component with a varying concentration. 

It appeared that the best agreement between the values of the surface tension of the TX165 aqueous solutions with the RL and SF mixture calculated from Equation (2) and those measured is obtained if the value of the surface tension of the mixture component at the constant concentration and the values of the variable concentrations of the other mixture component are applied in this equation (Figures S1, S4, S7). Unfortunately, the $\gamma_{LV}$ isotherms for the aqueous solution of the TX165 + RL and TX165 + SF mixtures at the constant concentration of one component and variable of the other can be described by Equation (2) if the constant concentration values are smaller than $C_{min}^{sat}$.

On the other hand, it was possible to describe all $\gamma_{LV}$ isotherms for the aqueous solutions of the studied binary mixtures of surface-active compounds with the constant composition and the variable concentration (Figures S18–S25). Analyzing the $n\Gamma^{max}$ and a values used for the calculation of the surface tension of the aqueous solution of the binary mixtures of TX165 with the biosurfactants, it can be concluded that the $n\Gamma^{max}$ values change almost linearly with the mixture composition. Moreover, in the case of the a value, a negative deviation from the linear dependence on the mixture composition is observed (Figure S26). It can be stated that using the Szyszkowski equation [27], it is possible not only to describe but also to predict the $\gamma_{LV}$ isotherms based on the data of the particular component of the surfactant mixtures if the relationship between these data and the mixture composition is known. 

From the theoretical and practical points of view, it is more important to predict the surface tension of the aqueous solution of surfactant mixtures than only to describe it. Among the concepts which can be used for the prediction of $\gamma_{LV}$ values for the aqueous solutions of surfactant mixtures, the one proposed by Fainerman and Miller seems to be very useful [34,35]. However, while using the Fainerman and Miller concept for the determination of the surface tension of the aqueous solution of surfactant mixtures, the main problem is to establish the values of the area occupied by the mole of each component of the mixture as well as some average values for surfactant mixtures (ϖ). The ϖ of the surfactants and their mixtures is equal to $\frac{1}{\Gamma^{\infty }}$, where $\Gamma^{\infty }$ is the limiting concentration of a given component of the surfactant mixtures or mixture in the monolayer at the water–air interface. $\Gamma^{\infty }$ is equal to $\frac{1}{NA_{0}}$, where N is the Avogadro number and $A_{0}$ is the limiting area occupied by one surfactant molecule. Assuming that the surfactant molecules at their limiting concentration in the monolayer at the water–air interface are oriented perpendicular to the interface, the $A_{0}$ value can be determined based on the bond length between the atoms in the surfactant molecule, the angle between the bonds, and the average allowed distance. The values of $A_{0}$ for TX165, RL and SF calculated in this way are close to 35.70, 69.09, and 93.17 Å^2^, respectively [22,23]. Knowing the $A_{0}$ value of a given surfactant in the mixture, it is easy to calculate its ϖ value. However, the main problem is calculating the ϖ values for the surfactant mixtures. If the $A_{0}$ values of particular components of the mixture are the same or close, the determination of ϖ for the surfactant mixtures is easy. However, in our case, there are great differences between the $A_{0}$ values of TX165, RL, and SF. It seems reasonable to assume that the $\Gamma_{12}^{\infty }$ of the TX165 mixtures with RL or SF is equal to $\Gamma_{1}^{\infty }x_{1}^{S}+\Gamma_{2}^{\infty }x_{2}^{S}$ where $x^{S}$ is the mole fraction of the particular surfactants in the mixture and 1, 2, and 12 refer to TX165, the biosurfactant, and the mixture of TX165 with the biosurfactant, respectively. As was stated earlier [33], $x_{1}^{S}=\frac{\pi_{1}}{\pi_{1}+\pi_{2}}$ and $x_{2}^{S}=\frac{\pi_{2}}{\pi_{1}+\pi_{2}}$ ($\pi_{1}$ and $\pi_{2}$ are the layers surfactants 1 and 2 pressure, respectively). Taking into account the $\Gamma_{12}^{\infty }$ values determined in this way, the ϖ values for the TX165 + RL and TX165 + SF mixtures at a given concentration and composition were deduced. Knowing the ϖ values for the mixtures and particular components, the surface tension of the aqueous solution of TX165 mixtures with RL and SF was calculated from the Fainerman and Miller equation, which for the binary mixtures has the form [27,28]:

(3) $$\exp\prod =\exp\prod_{1}+\exp\prod_{2}-1\;$$

where ∏=πϖ/RT, $\prod_{1}=\pi_{1}\varpi_{1}/RT$ and $\prod_{2}=\pi_{2}\varpi_{2}/RT$ (*R* is the gas constant and *T* is the temperature).

It appeared that based on Equation (3) it was possible to predict the surface tension for the aqueous solution of the TX165 mixture with RL or SF if the constant concentration of one component of the mixture was smaller than its $C_{min}^{sat}$ in the whole variable concentration of the other mixture component (Figures S1, S4, S7, S10). For the aqueous solution of the TX165 and biosurfactant mixture at the concentration of both mixture components higher than their $C_{min}^{sat}$, the agreement between the measured and calculated (from Equation (3)) values of surface tension is observed only at some mixture concentrations (Figures S3, S6, S9, S12). In the case of the TX165 mixtures with biosurfactants at a constant composition, the agreement between the values of the measured and calculated (from Equation (3)) surface tension is not observed in the whole range of mixture concentrations. It is possible that due to the stronger interactions between the TX165 molecules and the biosurfactant compared to that between the molecules of the same compound, the surface area occupied by a mole of the mixture is different from that calculated. Such a conclusion is based on the fact that with the hydrophilic part of the TX165, the H_3_O^+^ ions can be joined by the hydrogen bonds and the nonionic surfactant can be treated as the cationic one [27]. Therefore the electrostatic interactions can take place between TX165 and the biosurfactants. It seems, however, that despite the strong interactions between the TX165 molecules and those of biosurfactants, the mole fraction of the mixture components in the mixed monolayer at the water–air interface does not differ much from that calculated based on the monolayer pressure of individual compounds. This conclusion confirms the surface tension isotherms of the aqueous solution of TX165 mixtures with the biosurfactants determined from the following expression [33]:

(4) $$\gamma_{LV}=\gamma_{LV}^{1}x_{1}^{S}+\gamma_{LV}^{2}x_{2}^{S}$$

It appeared that for the most studied aqueous solutions of the binary mixtures, the surface tension can be predicted from Equation (4) (Figures S1–S12, S18–S25). The values of $\gamma_{LV}$ calculated from Equation (4) confirmed that maxima on the $\gamma_{LV}$ isotherms are possible.

### 2.3. Concentration and Composition of the Mixed Monolayer

Based on the $\gamma_{LV}$ isotherms of the aqueous solution of the single surfactants and their mixtures, it is possible to determine the surface concentration of a given surfactant or biosurfactant in both the individual and mixed monolayers at the water–air interface. For this purpose, the Gibbs isotherm equation is most often used for both the aqueous solution of individual surfactants and their mixture, in which the concentration of one component is variable but that of the other is constant, or for the mixtures at a constant composition and variable total concentration.

The Gibbs equation for the aqueous solution of multi-component surfactant mixtures has the form [27]:

(5) $$\Gamma =-\frac{a_{i}}{nRT}{\left(\frac{\partial \gamma_{LV}}{\partial a_{i}}\right)}_{i\neq j,T}=-\frac{C_{i}}{nRT}{\left(\frac{\partial \gamma_{LV}}{\partial C_{i}}\right)}_{i\neq j,T}=-\frac{1}{2.303nRT}{\left(\frac{\partial \gamma_{LV}}{\partial \log C_{i}}\right)}_{i\neq j,T}$$

Using Equation (5) for the calculation of the surfactant concentration in the monolayer or the mixed monolayer at the water–air interface, its limitations should be kept in mind. If for the calculation of *Γ*, the concentration of surfactants in mole/dm^3^ is applied, then it is assumed that the coefficient of the surfactant activity is equal to 1 and the mole fraction of the surfactant is equal to $\frac{C_{i}}{\omega }$, where *ω* is the number of the water moles in 1 dm^3^ at a given temperature. As a matter of fact, the concentration of the surfactants and their mixture is so small that it is not taken into account in the *ω* calculation. It should also be remembered that *Γ* is not the total concentration of the surfactants in the monolayer but the so-called Gibbs surface excess concentration. However, the difference between the surfactants’ concentration in the surface region and in the bulk phase is so great that *Γ* can be treated as the total concentration.

For the aqueous solution of TX165 mixtures with RL and SF at the constant concentration of one component and variable of the other one, the isotherms of *Γ* can be determined in the whole range of variable concentrations of one component of the mixture only when the isotherms of $\gamma_{LV}$ can be described by one exponential function; in other words, only for the $\gamma_{LV}$ isotherms on which the maxima are not present. The *Γ* isotherms of TX165 calculated from Equation (5) at the constant concentration of RL and SF smaller than $C_{min}^{sat}$ have a shape similar to the *Γ* isotherm of single TX165 (exemplary Figure S27). On the other hand, the shape of the isotherms *Γ* for RL and SF at a constant concentration of TX165 is similar to that of individual biosurfactants (exemplary Figure S28). Since it is difficult to calculate the *Γ* isotherms for all tested systems applying Equation (5), conclusions about the interactions between the molecules of the components of the mixed saturated monolayer can hardly be drawn. However, it is possible to calculate the *Γ* isotherms from those of $\gamma_{LV}$ with extremes using the Frumkin equation [27,36]. 

Yet this is the main problem to solve the Frumkin equation against *Γ*. It has to do with the maximal concentration of each component of the mixture in the surface mixed monolayer at its given composition. It seems reasonable to assume that the maximum concentration in the mixed monolayer of each mixture component at its given concentration in the bulk phase can be approximately equal to the product of the fraction of the surface area occupied by that component and its individual maximum concentration ($x^{S}\Gamma^{max}$). On the other hand, the water surface tension reduction by the adsorption of a given component of the surfactant mixture at the water–air interface can be expressed by the difference in the surface tension of water ($\gamma_{W}$) and solution ($\gamma_{LV}$) multiplied by the mole fraction of this component in the surface layer $\left(\left(\gamma_{W}-\gamma_{LV}\right)x^{S}=\pi x^{S}\right)$. Thus the Frumkin equation can be written in the form:

(6) $$\pi =-RT\Gamma^{max}\ln\left(1-\frac{\Gamma }{x^{S}\Gamma^{max}}\right)$$

Taking Equation (6) into account, it was possible to calculate *Γ* for TX165 and RL as well as for TX165 and SF, even in the case where the maxima are present on the $\gamma_{LV}$ isotherms (Figures S29–S34). The total *Γ* for TX165 and RL or SF calculated from Equation (6) for the aqueous solution of TX165 with the biosurfactants mixture at the constant concentration of one component and variable of the other one in the range of the constant concentrations below $C_{min}^{sat}$ is close to the *Γ* calculated from Equation (5). However, for the aqueous solution of TX165 + RL and TX165 + SF at a constant composition, the differences between the values of *Γ* determined from Equations (5) and (6) are observed (Figures S35 and S36). There may be two reasons for that. One can refer to the value of n in Equation (5) used for calculations, which is connected to the anionic biosurfactants of the 1: 1 type electrolyte, but their molecules in the mixture cannot be completely dissociated and the *n* value used by us is not proper. The other reason may result from the fact that, as mentioned above, H_3_O^+^ can be joined with the oxyethylene groups in the hydrophilic part of TX165 molecules [37]. In such a case, RL and SF can be treated as nonionic surfactants. In the calculations of *Γ* for TX165 and the biosurfactants, there are the $x^{S}$ values, whose determination is based on the contribution of particular components of the TX165 with RL and SF mixtures to the water surface tension reduction. For the determination of the composition of the mixed monolayer at the water–air interface, the relationship between the $x^{S}$ values calculated from the contributions of the components to the reduction in the $\gamma_{W}$ and their mole fraction in the mixed monolayer is of significant importance. The relative composition of the saturated mixed monolayer is very often calculated from the Hua and Rosen equation of the following form [27,38]:

(7) $$\frac{{\left(x_{1}^{S}\right)}^{2}\ln\left(x_{1}^{b}C_{12}/x_{1}^{S}C_{1}\right)}{{\left(1-x_{1}^{S}\right)}^{2}\ln\left[\left(1-x_{1}^{b}\right)C_{12}/\left(1-x_{1}^{S}\right)C_{2}\right]}=1$$

where indices 1, 2, and 12 refer to TX165, RL, or SF and to the mixtures of TX165 with RL and/or SF, respectively, and *b* refers to the bulk phase. It proved that the values of $x_{1}^{S}$ and $x_{2}^{S}$ determined using Equation (7) are similar to those determined in the way mentioned above (Tables S1 and S2 and Figure S37 as an example). As follows from the calculations of $x_{1}^{S}$ and $x_{2}^{S}$, the mole fraction of RL and SF in the mixed saturated monolayer is higher than in the bulk phase. This can be more clearly seen in the case of SF than RL. Based on the concept of Hua and Rosen [38], it is possible to determine the parameter of intermolecular interactions in the saturated mixed monolayer ($\beta^{\sigma })$. The equation resulting from this concept has the form:

(8) $$\beta^{\sigma }=\frac{\ln\left(x_{1}^{b}C_{12}/x_{1}^{S}C_{1}\right)}{{\left(1-x_{1}^{S}\right)}^{2}}$$

The calculated values of $\beta^{\sigma }$ indicate that in the case of the TX165 + RL mixture, the synergetic effect in the reduction in water surface tension is more visible than that for the TX165 and SF mixture (Tables S1 and S2). For the TX165 and SF mixture, the $\beta^{\sigma }$ parameter changes from negative to positive values depending on its composition and the surface tension is taken into consideration (Table S2). However, the absolute values of $\beta^{\sigma }$ are close to zero. As mentioned above, the activity of SF adsorption at the water–air interface is much greater than that of TX165. TX165 can not influence the adsorption of SF to such an extent that the synergetic effect in the water surface tension reduction could take place.

### 2.4. CMC

From the practical point of view, the second important property of surfactants and biosurfactants is their ability to form micelles in the polar environment. The surfactant concentration at which micelles are formed, known as the critical micelle concentration (CMC), can be determined by many methods. Among them, the method based on the $\gamma_{LV}$ isotherms is often used. Since it was not possible to determine the CMC based on the $\gamma_{LV}$ isotherms over the whole range of constant concentration values of one component of the mixture of TX165 with RL or TX165 with SF and the variable second component, the CMC was determined only for the mixtures with a constant composition (Figure 6 and Figure 7). The negative deviation of CMC as a function of biosurfactant mole fractions in the mixture with TX165 is observed. 

The CMC values obtained from the $\gamma_{LV}$ isotherms were compared to those calculated for the ideal mixture of surfactants from the following equation [27]:

(9) $$\frac{1}{{\mathrm{CMC}}_{12}}=\frac{x_{1}^{b}}{{\mathrm{CMC}}_{1}}+\frac{1-x_{1}^{b}}{{\mathrm{CMC}}_{2}}$$

where ${\mathrm{CMC}}_{1}$, ${\mathrm{CMC}}_{2}$, and ${\mathrm{CMC}}_{12}$ are the critical micelle concentrations of TX165, RL, and SF and their mixtures, respectively. 

An insignificant difference between the values of CMC for the mixtures of TX165 with the biosurfactants calculated from Equation (9) and those determined from the $\gamma_{LV}$ isotherms was found. Based on the comparison of CMC values calculated from Equation (9) and those determined from the $\gamma_{LV}$ isotherms, it cannot be explicitly stated whether there is a synergistic effect in the aggregation process of the mixed micelles of TX165 with the biosurfactants. Bergström and Eriksson [39] carried out studies on the synergistic effect in the micellization process of the surfactants’ binary mixtures. Based on the Poisson–Boltzman theory, they proposed an equation for the calculation of the CMC of the surfactant mixtures. The equation derived by them for the calculation of CMC for the nonionic and ionic surfactant mixtures has the form:

(10) $${\mathrm{CMC}}_{12}\left(x_{2}^{M}\right)={\left(x_{2}^{M}\right)}^{2}\exp\left(1-x_{2}^{M}\right){\mathrm{CMC}}_{2}+\left(1-x_{2}^{M}\right)\exp\left(1-x_{2}^{M}\right){\mathrm{CMC}}_{1\;\;\;\;\;\;\;\;\;\;\;\;\;\;\;\;}$$

where the $x_{2}^{M}$ is the mole fraction of the given component surfactant mixture in the micelles. The mole fraction of the surfactant mixture compounds can be determined from the Hua and Rosen equation [27,38]:

(11) $$\frac{{\left(x_{1}^{M}\right)}^{2}\ln(x_{1}^{b}{\mathrm{CMC}}_{12}/x_{1}^{M}{\mathrm{CMC}}_{1})}{{\left(1-x_{1}^{M}\right)}^{2}\ln\left[\left(1-x_{1}^{b}\right){\mathrm{CMC}}_{12}/\left(1-x_{1}^{M}\right){\mathrm{CMC}}_{2}\right]}=1$$

Knowing the mole fraction of TX165 and biosurfactant calculated from Equation (11), it is possible to calculate the parameter of intermolecular interactions in the micelle ($\beta^{M}$) [27,38]:

(12) $$\beta^{M}=\frac{\ln\left(x_{1}^{b}{\mathrm{CMC}}_{12}/x_{1}^{M}{\mathrm{CMC}}_{1}\right)}{\;{\left(1-x_{1}^{M}\right)}^{2}}$$

The $\beta^{M}$ values calculated from Equation (12) are negative. This indicates synergetic effects in the micelle formation for the TX165 + RL and TX165 + SF mixtures (Table S3). However, the latter condition should confirm this conclusion. As follows, $\left|\beta^{M}\right|>\left|\ln\left({\mathrm{CMC}}_{2}/{\mathrm{CMC}}_{1}\right)\right|$. This condition is also fulfilled for the TX165 with SF mixtures at mole fractions of SF equal to 0.4, 0.6, and 0.8. In the case of the TX165 + RL mixture, the existence of the synergetic effect in the micelle formation was confirmed by the latter condition only at the mole fractions of RL in the bulk phase, equal to 0.2 and 0.8. Based on the mole fraction of TX165 and biosurfactants, it is possible to calculate the CMC of the studied mixtures. As follows from the calculations, the values of CMC obtained from Equation (10) for the mixtures of TX165 with RL are close to those determined from the $\gamma_{LV}$ isotherm (Figure S38). In the case of the TX165 and SF mixture, an insignificant difference between these values is observed (Figure S39).

Taking into account the mole fraction of the TX165 and biosurfactants in the mixture, it is possible to calculate the coefficients of TX165 and biosurfactants activity in the mixed micelles from the following expressions [40]:

(13) $$\ln f_{1}^{M}=\beta^{M}{\left(1-x_{1}^{M}\right)}^{2}$$

(14) $$\ln f_{2}^{M}=\beta^{M}{\left(x_{1}^{M}\right)}^{2}$$

Knowing the $\ln f_{1}$ and $\ln f_{2}$ values, the CMC of the TX165+RL and TX165+SF mixtures can be calculated from the equation [27]:

(15) $$\frac{1}{{\mathrm{CMC}}_{12}}=\frac{x_{1}^{b}}{f_{1}{\mathrm{CMC}}_{1}}+\frac{1-x_{1}^{b}}{f_{2}{\mathrm{CMC}}_{2}}$$

The values of ${\mathrm{CMC}}_{12}$ calculated from Equation (15) for the mixtures of TX165 with RL and SF are close to those determined from the isotherms of the surface tension of their aqueous solutions (Figures S38 and S39).

The compatibility of the CMC values of TX165 mixtures with the biosurfactants determined based on the surface tension of their aqueous solutions with those calculated from Equations (9) and (15) does not indicate the synergistic effect in the micellization process.

On the other hand, the parameter of intermolecular interactions in the micelles determined from the Hua and Rosen theory satisfies, although not in every composition of mixtures, the two conditions for the synergistic effect in the micellization process.

It seems that the lack of reliable evidence of the synergetic effect in the micellization process may result from very great differences in the CMC values of individual components of the mixture, and the theories were proposed for the systems with smaller differences in their surface and volumetric properties.

### 2.5. Standard Gibbs Free Energy of Adsorption and Micellization

The standard free energy of adsorption$\;(\Delta G_{ads}^{0}$) and micellization $(\Delta G_{amic}^{0}$) is a measure of the surfactants and their mixture tendency to adsorb or aggregate in aqueous media. The literature reports many different methods for $\Delta G_{ads}^{0}$ determination [27]. Among them, the method based on the constant *a* is very often applied. The constant *a* can be determined, among others, by the Szyszkowski and linear Langmuir equations [27]. The dependence between the constant *a* and $\Delta G_{ads}^{0}$ has the form:

(16) $$a=\varpi \exp\frac{\Delta G_{ads}^{0}}{RT}$$

Using the Szyszkowski equation, it is possible to describe the $\gamma_{LV}$ isotherms of the aqueous solutions of TX165 mixtures with biosurfactants, in which the value of the concentration of one component of the mixture was constant but smaller than $C_{min}^{sat}$ and the value of the other was variable, as well as for the mixtures at a constant composition. Thus the constant in this equation was taken into account in the calculation of $\Delta G_{ads}^{0}$. It appears that $\Delta G_{ads}^{0}$ for the individual solutions of TX165 and RL calculated based on the constant a from the Szyszkowski equation using Equation (16) are similar to those determined by the other methods [23,30,31]. However, for SF, $\Delta G_{ads}^{0}$ is smaller than that obtained from the Langmuir equation (Table 1). For the first series of solutions in which the concentration of one component of surfactant was constant and the other was variable, the values of $\Delta G_{ads}^{0}$ for TX165, RL, and SF in the TX165 + RL and TX165 + SF mixtures were close to those of $\Delta G_{ads}^{0}$ for these surfactants in their individual solutions. However, in the case of the aqueous solutions of the TX165 with RL and SF mixture at a constant composition and a variable total concentration, the nonlinear dependence between the $\Delta G_{ads}^{0}$ and the mole fraction of the biosurfactant in the mixture was obtained. (Figure S40). However, it turned out that the relationship between the $\Delta G_{ads}^{0}$ and concentration of the biosurfactant in the TX165+RL mixture can be described by the following equation:

(17) $$\Delta G_{ads}^{0}=x_{1}^{b}\Delta G_{ads,1}^{0}+x_{2}^{b}\Delta G_{ads,2}^{0}+G_{mix}^{E}$$

where $G_{mix}^{E}$ is the Gibbs free energy of surfactants mixing.

For the mixed monolayer of the TX165 mixture with RL or SF, the $G_{mix}^{E}$ can be calculated from the equation:

(18) $$G_{mix}^{E}=RT\left(x_{1}^{s}\ln f_{1}^{S}+x_{2}^{s}\ln f_{2}^{S}\right)$$

The activity coefficients of TX165 ($f_{1}$) and RL or SF ($f_{2}$) in the mixed monolayer at the water–air interface were determined from the following expressions (Tables S1 and S2) [40]:

(19) $$\ln f_{1}^{S}=\beta^{\sigma }{\left(1-x_{2}^{s}\right)}^{2}$$

and

(20) $$\ln f_{2}^{S}=\beta^{\sigma }{\left(x_{1}^{s}\right)}^{2}$$

In the case of the TX 165+SF mixture, a greater difference between $\Delta G_{ads}^{0}$ calculated from Equations (16) and (17) is found. This may be a result of great differences in the adsorption activity between TX165 and SF and their molar fractions in the saturated monolayer determined from the Hua and Rosen equation [27,38].

The standard Gibbs free energy of micellization was determined only for the aqueous solution of the TX165 mixture with RL and/or SF in which the composition was constant but the total concentration was variable. For determination of $\Delta G_{mic}^{0}$, we used the following equation [27]:

(21) $$\Delta G_{mic}^{0}=RT\ln\frac{\mathrm{CMC}}{\omega }$$

The values of $\Delta G_{mic}^{0}$ calculated from Equation (21) do not change linearly as a function of the mole fraction of biosurfactants in the bulk phase. Some binary mixtures of surfactants can be predicted from the $\Delta G_{mic}^{0}$ components, their mole fraction in the mixture, and the Gibbs free energy of surfactants mixing in the micelle ($G_{mix}^{E,m}$) [40], according to the following equation:

(22) $$\Delta G_{mic}^{0}=x_{1}^{b}\Delta G_{mic,1}^{0}+x_{2}^{b}\Delta G_{mic,2}^{0}+G_{mix}^{E,m}$$

The $G_{mix}^{E,m}$ fulfils the equation [27,41]:

(23) $$\Delta G_{mix}^{E,m}=RT\left(x_{1}^{M}\ln f_{1}^{M}+x_{2}^{M}\ln f_{2}^{M}\right)$$

If the mole fractions of TX165, SF, and RL for the mixtures of TX165+RL and TX165+SF in the bulk phase were used in Equation (22), the calculated values of $\Delta G_{mic}^{0}$ were higher than those calculated from Equation (21) (Figures S41 and S42). If the values of $x_{1}^{M}$ and $x_{2}^{M}$ were used in Equation (22) instead of $x_{1}^{b}$ and $x_{2}^{b}$, the $\Delta G_{mic}^{0}$ values calculated from Equation (22) were closer to those calculated from Equation (21) than in the case of $x_{1}^{b}$ and $x_{2}^{b}$ application. This fact proves the presence of a synergistic effect in the micellization process of tested mixtures.

## 3. Materials and Methods

Triton X-165 (TX165) ((p- (1,1,3,3-tetramethylbutyl)-phenoxypolyoxyethylene glycol) of a purity over 99% was purchased from FLUKA (Steinheim, Germany). R-95 Rhamnolipid (95%) (RL) and surfactin (≥98%) (SF) were purchased from Sigma-Aldrich (Steinheim, Germany). TX165, RL, and SF were used for the aqueous solution preparation without further purification. Six series of solutions were prepared for the surface tension measurements. The first series included the aqueous solutions of the RL and TX165 mixture with the constant RL concentration, the values of which ranged from 2 × 10^−4^ to 40 mg/dm^3^, and the variable concentration of TX165 from 1 × 10^−8^ to 4 × 10^−3^ mole/dm^3^. The second series included the solution in which the concentration of TX165 was constant (in a range from 1 × 10^−8^ to 4 × 10^−3^ mole/dm^3^) and RL variable, from 2 × 10^−4^ to 40 mg/dm^3^. The third series included the aqueous solutions of the RL mixture with TX165 in which the RL concentration varied from 2 × 10^−4^ to 40 mg/dm^3^. The TX165 mixture was selected so that the molar fractions of TX165 in the mixture were 0.2, 0.4, 0.6, and 0.8. In other words, these were the aqueous solutions of RL and TX165 with a constant composition and variable concentrations. The fourth, fifth, and sixth series were the solutions of the mixture of SF and TX165 of the same concentration as those of the first, second, and third series for the RL and TX 165 mixture. All solutions were prepared using doubly distilled and deionized water (Destamat Bi18E) at an internal specific resistance of 18.2 × 10^6^ Ω⋅m. The water purity was additionally controlled by the surface tension measurements before the solutions’ preparation.

The surface tension ($\gamma_{LV}$) of the aqueous solution of rhamnolipid and TX165, as well as the surfactin and TX165 mixtures, was measured by the Krüss K9 tensiometer according to the platinum ring detachment method (du Nouy’s method) at 293 K. Before the surface tension measurements, the tensiometer was calibrated using water ($\gamma_{LV}$ = 72.8 mN/m) and methanol ($\gamma_{LV}$ = 22.5 mN/m). A more detailed procedure for measuring the surface tension was given earlier [23]. For each concentration of the aqueous solution of RL and TX165, as well as the TX165 and SF mixtures, the surface tension measurements were repeated at least ten times. The standard deviation was ±0.1 mN/m and the uncertainty of the surface tension measurements was in a range from 0.3% to 0.7%.

## 4. Conclusions

From the measurements of the surface tension of the aqueous solutions of the TX165 + RL and TX165 + SF mixtures at a constant concentration of one mixture component and a variable concentration of the other, it results that maxima are present on the obtained surface tension isotherms, but they are not observed on the surface tension isotherms at the constant mixture composition. The maxima are observed at the constant concentration value of one component mixture close or higher to the CMC. This phenomenon was explained based on the contribution of particular components of the mixture to the reduction in water surface tension. This is important not only from the theoretical but also from the practical point of view.

The isotherms of the surface tension of the aqueous solution of the TX165 with RL or SF mixtures at the constant composition and variable total concentration can be described by the exponential function of the second order and the Szyszkowski equation. The description of the $\gamma_{LV}\;$isotherms of the aqueous solution of the binary mixture of the surfactants by the Szyszkowski equation is a theoretical novelty. 

In most cases, the isotherms of the surface tension of the aqueous solution of TX165 + RL and TX165 + SF, on which the maxima are present, can be described by two exponential functions of the second order, one in the range concentration of the mixture component whose concentration is variable from zero to the value corresponding to the maximum of the surface tension, and the other in the concentration range above this, at which the maximum is observed.

The relationship between the constants in the equation of the exponential function of the second order, as well as the components and parameters of the surfactants and biosurfactants tail and head, the surface tension is not excluded.

The isotherms of the surface tension of the aqueous solution of TX165 + RL and TX165 + SF can be predicted by the Fainerman and Miller equation, except for the mixtures in which the concentration of one or two components corresponds to the saturated monolayer at the water–air interface of the aqueous solution of the mixture single components. The area occupied by one mole of the mixtures at the water–air interface can be deduced based on the contribution of the mixture-given component to the reduction in the water surface tension.

The composition of the mixed monolayer at the water–air interface, as well as the isotherm of the surface tension, can be predicted from the isotherms of the surface tension of the aqueous solution of individual components of the mixture. The prediction of the composition of the mixed monolayer at the water–air interface by means of the simple way proposed by us is comparable to that of the Hua and Rosen equation. Our concept of the composition of the mixed monolayer at the water–air interface determination can be used for the mixture in the concentration range from 0 to CMC in contrast to the Hua and Rosen concept, which is applicable in the concentration range corresponding to the saturated monolayer and in the range of the limited composition of the mixture in the bulk phase. 

Using the Hua and Rosen concept, the synergetic effect in the water surface tension reduction was deduced. This effect does not occur in the whole range of the TX165 + RL and TX165 + SF concentrations and is more visible for the TX165 and RL mixture than for the TX165 + SF mixture.

The synergetic effect in the CMC of the studied mixtures was also found using the Hua and Rosen concept.

Taking into account the mole fraction of the given component in the mixed monolayer and its maximal concentration in this monolayer in the Frumkin equation, it is possible to determine isotherms of particular components’ adsorption of the studied mixtures as well as the summary concentration.

The changes of the CMC of the TX165 + RL and TX165 + SF mixtures as a function of the biosurfactants mole fraction in the bulk phase can be determined based on the CMC particular components of the mixture and its composition.

The standard Gibbs free energy of the adsorption and micellization of the TX165 + RL does not change linearly as a function of the biosurfactant molar fraction in the mixture in the bulk phase. This energy depends not only on the Gibbs free energy of each component of the studied mixtures but also on their Gibbs free energy of mixing in the mixed monolayer and micelles, respectively.

## Figures and Tables

**Table 1:** The different physicochemical properties of water, RL, SF, and TX165.

Properties	Water	TX165	Rhamnolipid	Surfactin
M [g]	18.016	911.000	504.000	1036.340
$V_{molecule}$ [Å^3^] *	29.885	1581.170	779.400	1460.440 1739.006 1562.950
$V_{mole}$ [cm^3^] *	17.999	952.339	469.433	879.623 1047.403 941.365
$S_{contactable}$ [Å^2^] *	58.43	1411.58 363.29	586.59 304.08	1389.02 346.10
$\gamma_{LV}^{min}$ [mN/m]	−	39.50	27.89	32.37
$\Gamma^{max}$ [mol/m^2^]	−	2.12 × 10^−6^	2.01 × 10^−6^	1.38 × 10^−6^
$\Gamma^{\infty }$ [mol/m^2^]	16.600 × 10^−6^	4.650 × 10^−6^	2.403 × 10^−6^	1.782 × 10^−6^
$\frac{\Gamma^{max}}{\Gamma^{\infty }}$*	−	0.4559	0.8365	0.7744
$A^{min}$ [Å^2^]	−	78.32	82.60	93.17 120.24
$A^{0}$ [Å^2^]	10.00	35.70	69.09	93.17
$C_{min}^{sat}$ [M]	−	5.00 × 10^−5^	1.98 × 10^−6^	9.65 × 10^−8^
CMC [M]	−	5.41 × 10^−4^	5.21 × 10^−5^	9.66 × 10^−6^
$\Delta G_{ads}^{0}$ [kJ/mol] *	−	−44.00	−43.55	−46.22 −51.23
$\Delta G_{mic}^{0}$ [kJ/mol] *	−	−28.10	−33.80	−37.90
$\gamma_{LV}$ of tail [mN/m]	72.80	22.00	21.80	24.70
$\gamma_{LV}$ of head [mN/m]	72.80	35.84	38.39	42.80
$\gamma^{LW}$ of head [mN/m]	26.85	27.70	35.38	34.25
$\gamma^{AB}$ of head [mN/m]	45.95	8.14	3.01	8.55
$\gamma^{+}$ of head [mN/m]	22.975	0.33	0.04	0.37
$\gamma^{-}$ of head [mN/m]	22.975	50.20	56.74	49.39

* $V_{molecule}$, $V_{mole}$, $S_{contactable}$, $\frac{\Gamma^{max}}{\Gamma^{\infty }}$, $\Delta G_{ads}^{0}$, and $\Delta G_{mic}^{0}$ were calculated. The other parameters were taken from the literature [22,23,24].

## Data Availability

Not applicable.

## Supplementary Materials

The following supporting information can be downloaded at: https://www.mdpi.com/article/10.3390/molecules27113600/s1, Table S1: The values of the mole fraction of the surfactants in the mixed monolayer ($x_{1}^{S}-$TX165, $x_{2}^{S}-$RL), parameter of intermolecular interaction ($\beta^{\sigma }$), activity coefficients ($f_{1}^{S}$ and $f_{2}^{S}$), and Gibbs excess free energy of mixing ($G_{mix}^{E}$); Table S2: The values of the mole fraction of the surfactants in the mixed monolayer ($x_{1}^{S}-$ TX165, $x_{2}^{S}-$ SF), parameter of intermolecular interaction ($\beta^{\sigma }$) activity coefficients ($f_{1}^{S}$ and $f_{2}^{S}$), and Gibbs excess free energy of mixing ($G_{mix}^{E}$); Table S3: The values of the mole fraction of the surfactants in the mixed micelle ($x_{1}^{M}$- TX165, $x_{2}^{M}-$ RL or SF), parameter of intermolecular interaction ($\beta^{M}$), activity coefficients ($f_{1}^{M}$ and $f_{2}^{M}$) and Gibbs excess free energy of mixing ($G_{mix}^{E,m}$). Figure S1: A plot of the surface tension ($\gamma_{LV}$) of the aqueous solution of RL and TX165 mixture at the constant RL concentration equal to 0.00625 mg/dm^3^ vs. the logarithm of the TX165 concentration ($C_{\mathrm{TX}165}$) (a) and the logarithm of the total concentration of the TX165 + RL mixture ($C_{12}$) (b). Figure S2: A plot of the surface tension ($\gamma_{LV}$) of the aqueous solution of RL and TX165 mixture at the constant RL concentration equal to 5 mg/dm^3^ vs. the logarithm of the TX165 concentration ($C_{\mathrm{TX}165}$) (a) and the logarithm of the total concentration of the TX165 +RL mixture ($C_{12}$) (b); Figure S3: A plot of the surface tension ($\gamma_{LV}$) of the aqueous solution of the RL and TX165 mixture at the constant RL concentration equal to 40 mg/dm^3^ vs. the logarithm of the TX165 concentration ($C_{\mathrm{TX}165}$) (a) and the logarithm of the total concentration of the TX165 + RL mixture ($C_{12}$) (b); Figure S4: A plot of the surface tension ($\gamma_{LV}$) of the aqueous solution of the SF and TX165 mixture at the constant SF concentration equal to 0.00625 mg/dm^3^ vs. the logarithm of the TX165 concentration ($C_{\mathrm{TX}165}$) (a) and the logarithm of the total concentration of the TX165 + SF mixture ($C_{12}$) (b); Figure S5: A plot of the surface tension ($\gamma_{LV}$) of the aqueous solution of the SF and TX165 mixture at the constant SF concentration equal to 5 mg/dm^3^ vs. the logarithm of the TX165 concentration ($C_{\mathrm{TX}165}$) (a) and the logarithm of the total concentration of the TX165 + SF mixture ($C_{12}$) (b); Figure S6: A plot of the surface tension ($\gamma_{LV}$) of the aqueous solution of the SF and TX165 mixture at the constant SF concentration equal to 40 mg/dm^3^ vs. the logarithm of the TX165 concentration ($C_{\mathrm{TX}165}$) (a) and the logarithm of the total concentration of the TX165 + SF mixture ($C_{12}$) (b); Figure S7: A plot of the surface tension ($\gamma_{LV}$) of the aqueous solution of the RL and TX165 mixture at a constant TX165 concentration equal to 5 × 10^−7^ mole/dm^3^ vs. the logarithm of the RL concentrations ($C_{\mathrm{RL}}$) (a) and the logarithm of the total concentration of the TX165 + RL mixture ($C_{12}$) (b); Figure S8: A plot of the surface tension ($\gamma_{LV}$) of the aqueous solution of the RL and TX165 mixture at a constant TX165 concentration equal to 2 × 10^−4^ mole/dm^3^ vs. the logarithm of the RL concentration ($C_{\mathrm{RL}}$) (a) and the logarithm of the total concentration of the TX165 + RL mixture ($C_{12}$) (b); Figure S9: A plot of the surface tension ($\gamma_{LV}$) of the aqueous solution of the RL and TX165 mixture at the constant TX165 concentration equal to 1 × 10^−3^ mole/dm^3^ vs. the logarithm of the RL concentration ($C_{\mathrm{RL}}$); Figure S10: A plot of the surface tension ($\gamma_{LV}$) of the aqueous solution of the SF and TX165 mixture at the constant TX165 concentration equal to 5 × 10^−7^ mole/dm^3^ vs. the logarithm of the SF concentration ($C_{\mathrm{SF}}$) (a) and the logarithm of the total concentration of the TX165 + SF mixture ($C_{12}$) (b); Figure S11: A plot of the surface tension ($\gamma_{LV}$) of the aqueous solution of the SF and TX165 mixture at the constant TX165 concentration equal to 2 × 10^−4^ mole/dm^3^ vs. the logarithm of the SF concentration ($C_{\mathrm{SF}}$); Figure S12: A plot of the surface tension ($\gamma_{LV}$) of the aqueous solution of the SF and TX165 mixture at the constant TX165 concentration equal to 1 × 10^−3^ mole/dm^3^ vs. the logarithm of the SF concentration ($C_{\mathrm{SF}}$); Figure S13: A plot of the constant $y_{0}$ in Equation (1) for the TX165 + RL (curve 1) and TX165 + SF (curve 2) aqueous solutions vs. the biosurfactant mole fraction in the mixture in the bulk phase ($x_{2}^{b}$); Figure S14: A plot of the constant $A_{1}$ in Equation (1) for the TX165 + RL (curve 1) and TX165 + SF (curve 2) aqueous solutions vs. the biosurfactant mole fraction in the mixture in the bulk phase ($x_{2}^{b}$); Figure S15: A plot of the constant $A_{2}$ in Equation (1) for the TX165 + RL (curve 1) and TX165 + SF (curve 2) aqueous solutions vs. the biosurfactant mole fraction in the mixture in the bulk phase ($x_{2}^{b}$); Figure S16: A plot of the constant $t_{1}$ in Equation (1) for the TX165 + RL (curve 1) and TX165 + SF (curve 2) aqueous solutions vs. the biosurfactant mole fraction in the mixture in the bulk phase ($x_{2}^{b}$); Figure S17: A plot of the constant $t_{2}$ in Equation (1) for the TX165 + RL (curve 1) and TX165 + SF (curve 2) aqueous solutions vs. the biosurfactant mole fraction in the mixture in the bulk phase ($x_{2}^{b}$); Figure S18: A plot of the surface tension ($\gamma_{LV}$) of the aqueous solution of the RL and TX165 mixture at the RL mole fraction equal to 0.2 vs. the logarithm of the total concentration of the TX165 + RL mixture ($C_{12})$; Figure S19: A plot of the surface tension ($\gamma_{LV}$) of the aqueous solution of the RL and TX165 mixture at the RL mole fraction equal to 0.4 vs. the logarithm of the total concentration of the TX165 + RL mixture ($C_{12})$; Figure S20: A plot of the surface tension ($\gamma_{LV}$) of the aqueous solution of the RL and TX165 mixture at the RL mole fraction equal to 0.6 vs. the logarithm of the total concentration of the TX165 + RL mixture ($C_{12})$; Figure S21: A plot of the surface tension ($\gamma_{LV}$) of the aqueous solution of the RL and TX165 mixture at the RL mole fraction equal to 0.8 vs. the logarithm of the total concentration of the TX165 + RL mixture ($C_{12})$; Figure S22: A plot of the surface tension ($\gamma_{LV}$) of the aqueous solution of the SF and TX165 mixture at the SF mole fraction equal to 0.2 vs. the logarithm of the total concentration of the TX165 + SF mixture ($C_{12})$; Figure S23: A plot of the surface tension ($\gamma_{LV}$) of the aqueous solution of the SF and TX165 mixture at the SF mole fraction equal to 0.4 vs. the logarithm of the total concentration of the TX165 + SF mixture ($C_{12})$; Figure S24: A plot of the surface tension ($\gamma_{LV}$) of the aqueous solution of the SF and TX165 mixture at the SF mole fraction equal to 0.6 vs. the logarithm of the total concentration of the TX165 + SF mixture ($C_{12})$; Figure S25: A plot of the surface tension ($\gamma_{LV}$) of the aqueous solution of the SF and TX165 mixture at the SF mole fraction equal to 0.8 vs. the logarithm of the total concentration of the TX165 + SF mixture ($C_{12})$; Figure S26: A plot of the constant a in the Szyszkowski equation (Equation (2)) for the TX165 + RL (curve 1) and TX165 + SF (curve 2) aqueous solutions vs. the biosurfactant mole fraction in the mixture in the bulk phase ($x_{2}^{b}$); Figure S27: A plot of the surface concentration (*Γ*) of TX165 (curves 1, 1′, 2, 2′), RL (curve 3), and SF (curve 5) vs. the logarithm of TX165 concentration ($C_{\mathrm{TX}165}$) at the constant biosurfactant concentration equal to 0.00625 mg/dm^3^; Figure S28: A plot of the surface concentration (*Γ*) of RL (curves 1, 1′), SF (curves 2, 2′), and TX165 (curves 3 and 5) vs. the logarithm of biosurfactant concentration (C) at the constant TX165 concentration equal to 5 × 10^−7^ mole/dm^3^; Figure S29: A plot of the surface concentration (*Γ*) of TX165 calculated from Equation (6) in the TX165 + RL mixture vs. the logarithm of its concentration ($C_{\mathrm{TX}165}$); Figure S30: A plot of the surface concentration (*Γ*) of TX165 calculated from Equation (6) in the TX165 + SF mixture vs. the logarithm of its concentration ($C_{\mathrm{TX}165}$); Figure S31: A plot of the surface concentration (*Γ*) of RL calculated from Equation (6) in the TX165 + RL mixture vs. the logarithm of TX165 concentration ($C_{\mathrm{TX}165}$); Figure S32: A plot of the surface concentration (*Γ*) of SF calculated from Equation (6) in the TX165 + SF mixture vs. the logarithm of TX165 concentration ($C_{\mathrm{TX}165}$); Figure S33: A plot of the total surface concentration (*Γ*) of the TX165 + RL mixture calculated from Equation (6) vs. the logarithm of TX165 concentration ($C_{\mathrm{TX}165}$); Figure S34: A plot of the total surface concentration (*Γ*) of the TX165 + SF mixture calculated from Equation (6) vs. the logarithm of TX165 concentration ($C_{\mathrm{TX}165}$); Figure S35: A plot of the total surface concentration (*Γ*) of the TX165 + RL mixture calculated from Equation (6) (curves 1, 2, 3, and 4) and Gibbs surface concentration calculated from Equation (5) vs. the logarithm of the total concentration of TX165 + RL mixture ($C_{12})$; Figure S36: A plot of the total surface concentration (*Γ*) of theTX165 + SF mixture calculated from Equation (6) (curves 1, 2, 3, and 4) and Gibbs surface concentration calculated from Equation (5) vs. the logarithm of the total concentration of TX165 + SF mixture ($C_{12})$; Figure S37: A plot of the TX165 mole fraction in the mixture with RL (curves 1 and 1′) and SF (curves 2 and 2′) (x) at the constant biosurfactant mole fraction in the mixture in the bulk phase equal to 0.2 vs. the total concentration of the TX165 + RL mixture ($C_{12}$); Figure S38: A plot of the CMC values of TX165 + RL and their mixtures vs. the RL mole fraction in the mixture in the bulk phase ($x_{2}^{b}$); Figure S39: A plot of the CMC values of TX165 + SF and their mixtures vs. the SF mole fraction in the mixture in the bulk phase ($x_{2}^{b}$); Figure S40: A plot of the Gibbs standard free energy of TX165 + RL (curves 1 and 1′) and TX165 + SF (curves 2 and 2′) adsorption at the water–air interface vs. the biosurfactant mole fraction in the mixture in the bulk phase ($x_{2}^{b}$); Figure S41: A plot of the Gibbs standard free energy of TX165 + RL micellization vs. the RL mole fraction in the mixture in the bulk phase ($x_{2}^{b}$) calculated from Equation (21) (curve 1) and form Equation (22) (curves 2 and 3); Figure S42: A plot of the Gibbs standard free energy of TX165 + SF micellization vs. the SF mole fraction in the mixture in the bulk phase ($x_{2}^{b}$) calculated from Equation (21) (curve 1) and form Equation (22) (curves 2 and 3).

## Sample Availability

Samples of the compounds are not available from the authors.
