# Supplementary material for: Thermodynamic Analysis of the Adsorption and Micellization Activity of the Mixtures of Rhamnolipid and Surfactin with Triton X-165
Source: Molecules. 2022 Jun 3;27(11):3600. doi: 10.3390/molecules27113600 (PMC9182474; doi:10.3390/molecules27113600)
Supplement: Supplementary file 1 [file molecules-27-03600-s001.zip › molecules-1736851-supplementary.pdf]

Table S1. The values of the the mole fraction of the surfactants in the mixed monolayer (x_1^S − TX165, x_2^S − RL), parameter of intermolecular interaction (β^σ), activity coefficients ($f_1^S$and f_2^S) and Gibbs excess free energy of mixing (G_{mix}^E).

γ_{LV} **[mN/m]**	x_1^S	x_2^S	β^σ	f_1^S	f_2^S	G_{mix}^E **[kJ/mol]**
			$x_2^b = 0.2$			
70	0.6607	0.3393	-2.0802	0.7870	0.4033	-1.1360
65	0.6654	0.3346	-2.3366	0.7698	0.3554	-1.2673
60	0.6539	0.3461	-1.3074	0.8550	0.5718	-0.7208
55	0.4152	0.5848	-2.3955	0.4408	0.6617	-1.4169
			$x_2^b = 0.4$			
70	0.5655	0.4345	-0.6910	0.8777	0.8017	-0.4136
65	0.5406	0.4594	-3.8980	0.4393	0.3201	-2.3582
60	0.5244	0.4756	0.8195	1.2036	1.2528	0.4979
55	0.3418	0.6582	-3.3947	0.2298	0.6726	-1.8604
			$x_2^b = 0.6$			
70	0.4189	0.5811	-0.7973	0.7640	0.8695	-0.4728
65	0.3984	0.6016	0.3979	1.1549	1.0652	0.2323
60	0.3472	0.6528	-0.3991	0.8436	0.9530	-0.2203
55	0.2105	0.7895	-2.1039	0.2694	0.9110	-0.8516
			$x_2^b = 0.8$			
70	0.2840	0.7160	-1.1865	0.5443	0.9088	-0.5877
65	0.2620	0.7380	-0.5808	0.7288	0.9609	-0.2736
60	0.2339	0.7661	-1.0286	0.5468	0.9453	-0.4489
55	0.1736	0.8264	-3.0037	0.1286	0.9135	-1.0497

Table S2. The values of the the mole fraction of the surfactants in the mixed monolayer (x_1^S − TX165, x_2^S − SF), parameter of intermolecular interaction (β^σ) activity coefficients ($f_1^S$and f_2^S) and Gibbs excess free energy of mixing (G_{mix}^E).

γ_{LV} **[mN/m]**	x_1^S	x_2^S	β^σ	f_1^S	f_2^S	G_{mix}^E **[kJ/mol]**
			$x_2^b = 0.2$			
70	0.3518	0.6482	0.3484	1.1576	1.0441	0.1935
65	0.2600	0.7400	0.6884	1.4578	1.0476	0.3226
60	0.1331	0.8669	0.9803	2.0890	1.0175	0.2756
55	0.0585	0.9415	-0.0932	0.9207	0.9997	-0.0125
			x_2^b =0.4			
70	0.1262	0.8738	0.5973	1.5779	1.0096	0.1604
65	0.1139	0.8861	0.4603	1.4353	1.0060	0.1131
60	0.0722	0.9278	0.4892	1.5237	1.0026	0.0798
55	0.0522	0.9478	-1.0516	0.3888	0.9971	-0.1267
			x_2^b =0.6			
70	0.1531	0.8469	-0.8491	0.5439	0.9803	-0.2682
65	0.0947	0.9053	-0.3074	0.7773	0.9972	-0.0642
60	0.0240	0.9760	0.7964	2.1353	1.0005	0.0455
55	0.0203	0.9797	-0.8086	0.4602	0.9997	-0.0391
			x_2^b =0.8			
70	0.0989	0.9011	-1.3348	0.3383	0.9870	-0.2898
65	0.0534	0.9466	-0.6864	0.5406	0.9980	-0.0845
60	0.0114	0.9886	0.5553	1.7207	1.0001	0.0152
55	0.0097	0.9903	-1.0278	0.3650	0.9999	-0.0241

Table S3. The values of the the mole fraction of the surfactants in the mixed micelle (x_1^M- TX165, x_2^M – RL or SF), parameter of intermolecular interaction (β^M), activity coefficients ($f_1^M$and f_2^M) and Gibbs excess free energy of mixing($G_{mix}^{E,m}$).

**TX165 + SF**						
x_2^b	x_1^M	x_2^M	β^M	f_1^M	f_2^M	$G_{mix}^{E,m}$ [kJ/mol]
0.2	0.2597	0.7403	-3.3116	0.1628	0.7998	-1.5509
0.4	0.2463	0.7537	-4.9288	0.0608	0.7416	-2.2286
0.6	0.2192	0.7808	-5.6293	0.0323	0.7629	-2.3474
0.8	0.1371	0.8629	-4.9207	0.0256	0.9117	-1.4177
**TX165 + RL**						
x_2^b	x_1^M	x_2^M	β^M	f_1^M	f_2^M	$G_{mix}^{E,m}$ (kJ/mol)
0.2	0.4017	0.5983	-2.8276	0.3634	0.6337	-1.6554
0.4	0.2374	0.7626	-1.4631	0.4270	0.9209	-0.6452
0.6	0.1492	0.8508	-1.4336	0.3543	0.9686	-0.4434
0.8	0.1369	0.8631	-2.5969	0.1445	0.9525	-0.7474

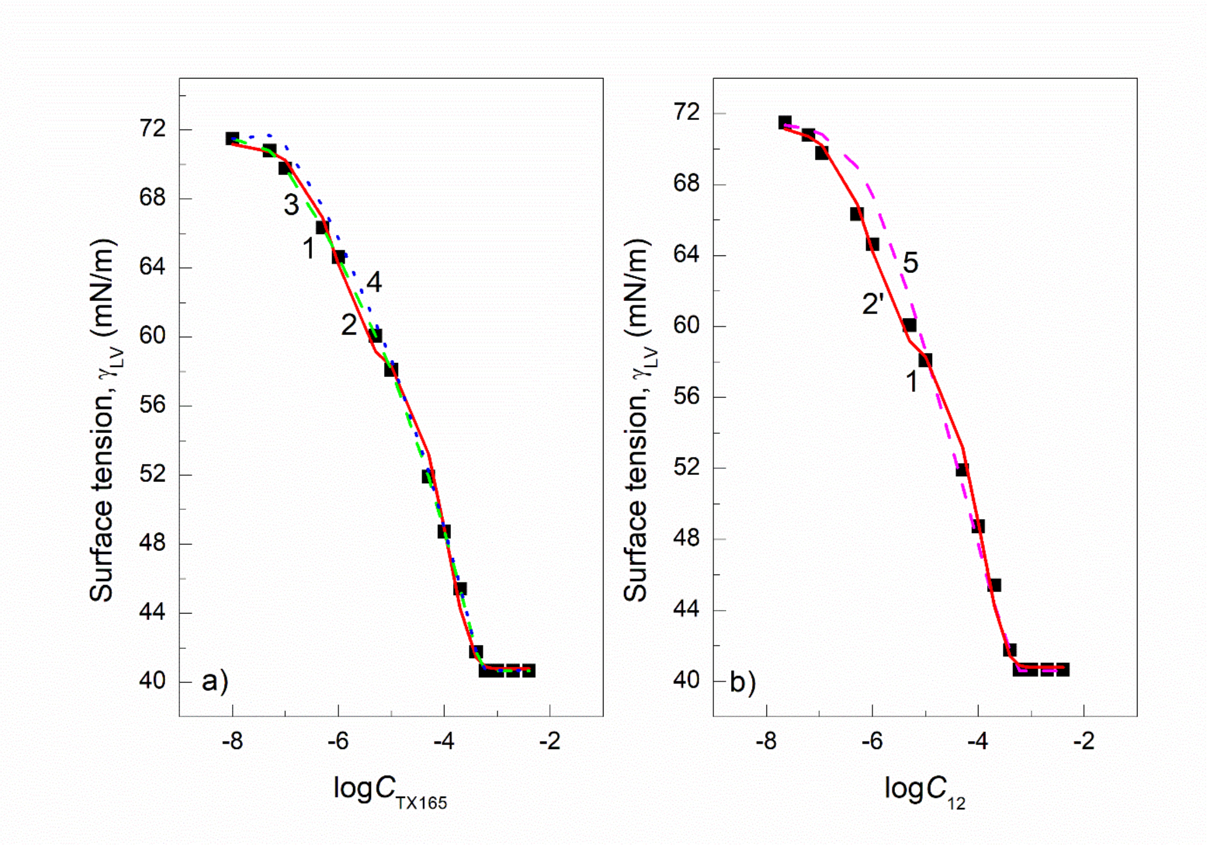

Figure S1. A plot of the surface tension (γ_{LV}) of the aqueous solution of RL and TX165 mixture at the constant RL concentration equal to 0.00625 mg/dm^3 vs. the logarithm of the TX165 concentration (C_{TX165}) (a) and the logarithm of the total concentration of the TX165 + RL mixture (C_{12}) (b). Points 1 correspond to the measured values. Curves 2 and 2′ correspond to the value calculated from Eq. (1), curves 3, 4 and 5 correspond to the values calculated from Eqs. (3), (4) and (2), respectively.

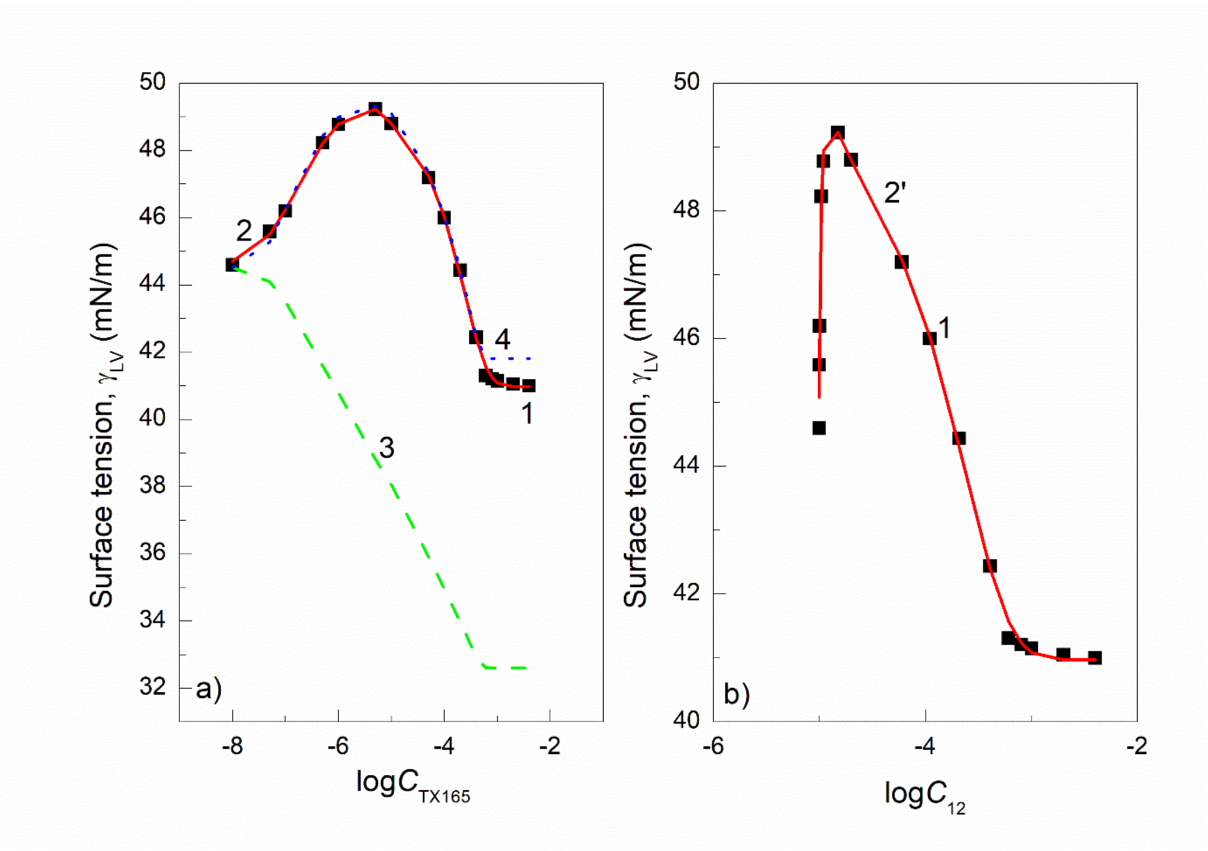

Figure S2. A plot of the surface tension (γ_{LV}) of the aqueous solution of RL and TX165 mixture at the constant RL concentration equal to 5 mg/dm^3 vs. the logarithm of the TX165 concentration (C_{TX165}) (a) and the logarithm of the total concentration of the TX165 + RL mixture (C_{12}) (b). Points 1 correspond to the measured values. Curves 2 and 2′ correspond to the values calculated from Eq. (1), curves 3 and 4 correspond to the values calculated from Eqs. (3) and (4), respectively.

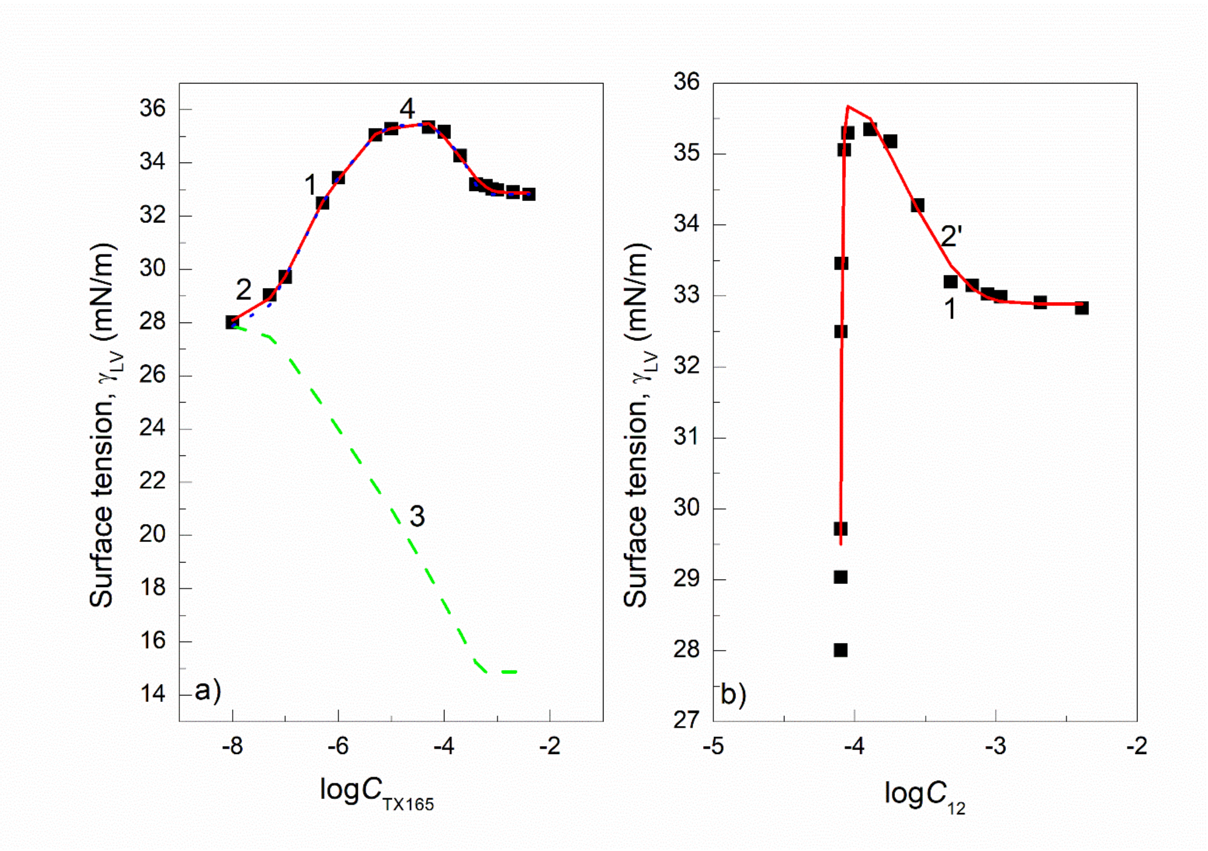

Figure S3. A plot of the surface tension (γ_{LV}) of the aqueous solution of RL and TX165 mixture at the constant RL concentration equal to 40 mg/dm^3 vs. the logarithm of the TX165 concentration (C_{TX165}) (a) and the logarithm of the total concentration of the TX165 + RL mixture (C_{12}) (b). Points 1 correspond to the measured values. Curves 2 and 2′ correspond to the values calculated from Eq. (1), curves 3 and 4 correspond to the values calculated from Eqs. (3) and (4), respectively.

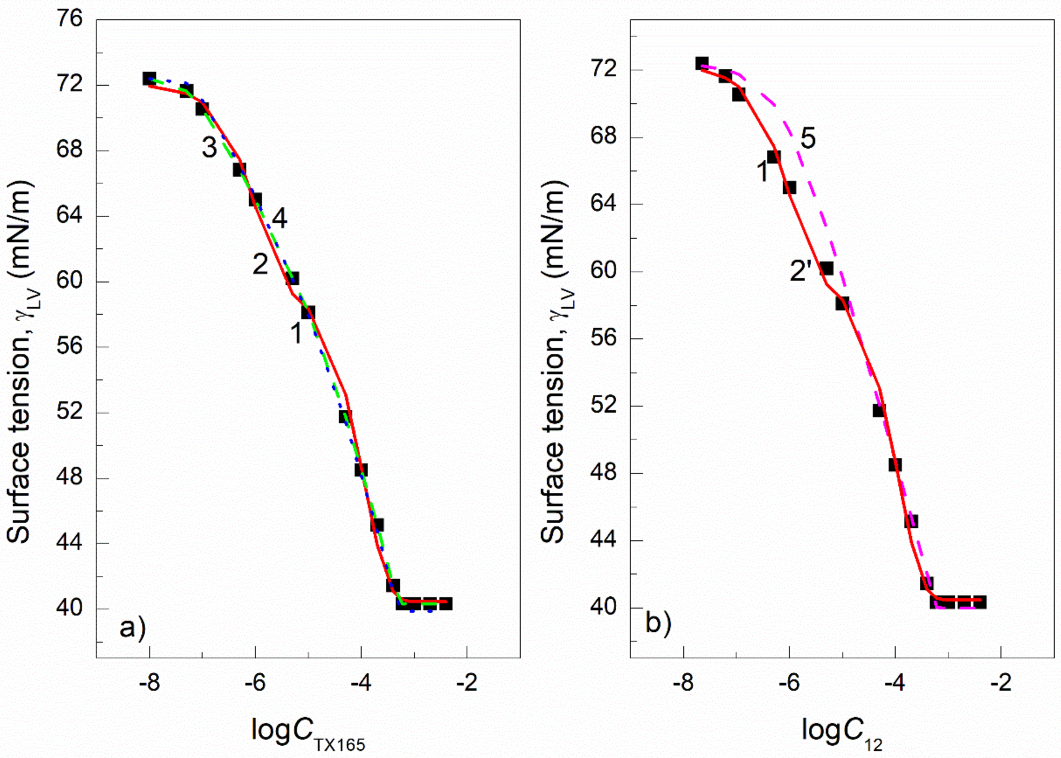

Figure S4. A plot of the surface tension (γ_{LV}) of the aqueous solution of SF and TX165 mixture at the constant SF concentration equal to 0.00625 mg/dm^3 vs. the logarithm of the TX165 concentration (C_{TX165}) (a) and the logarithm of the total concentration of the TX165 + SF mixture (C_{12}) (b). Points 1 correspond to the measured values. Curves 2 and 2′ correspond to the values calculated from Eq. (1), curves 3, 4 and 5 correspond to the values calculated from Eqs. (3), (4) and (2), respectively.

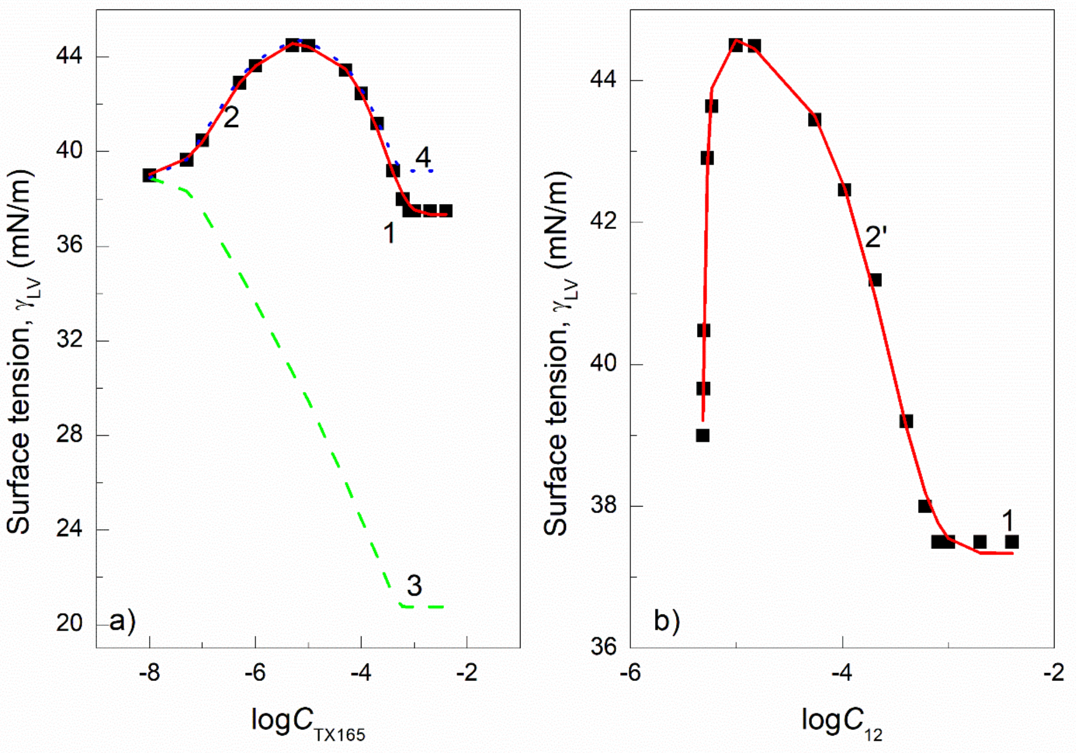

Figure S5. A plot of the surface tension (γ_{LV}) of aqueous solution of SF and TX165 mixture at the constant SF concentration equal to 5 mg/dm³ vs. the logarithm of the TX165 concentration (C_{TX165}) (a) and the logarithm of the total concentration of the TX165 + SF mixture (C_{12}) (b). Points 1 correspond to the measured values. Curves 2 and 2′ correspond to the values calculated from Eq. (1), curves 3 and 4 correspond to the values calculated from Eqs. (3) and (4), respectively.

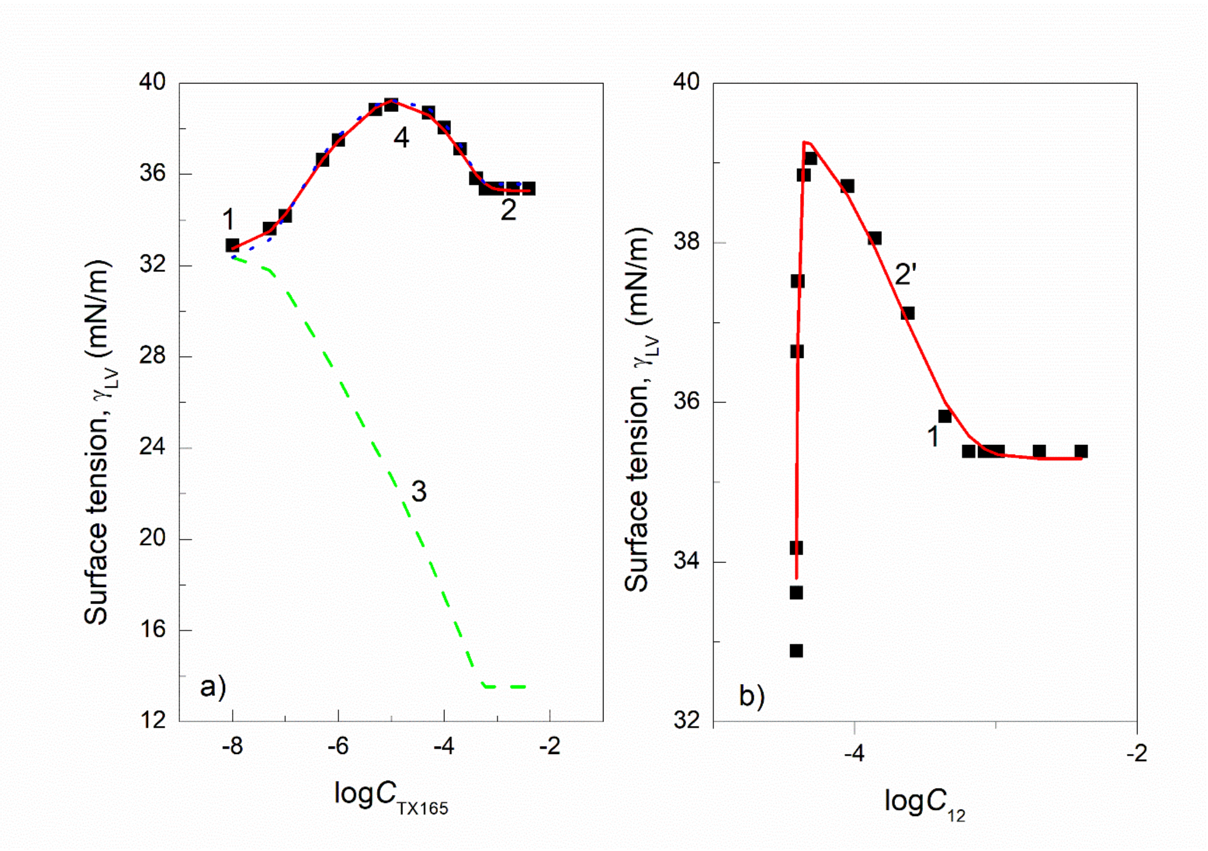

Figure S6. A plot of the surface tension (γ_{LV}) of the aqueous solution of SF and TX165 mixture at the constant SF concentration equal to 40 mg/dm[3] vs. the logarithm of the TX165 concentration (C_{TX165}) (a) and the logarithm of the total concentration of the TX165 + SF mixture (C_{12}) (b). Points 1 correspond to the measured values. Curves 2 and 2′ correspond to the values calculated from Eq. (1), curves 3 and 4 correspond to the values calculated from Eqs. (3) and (4), respectively.

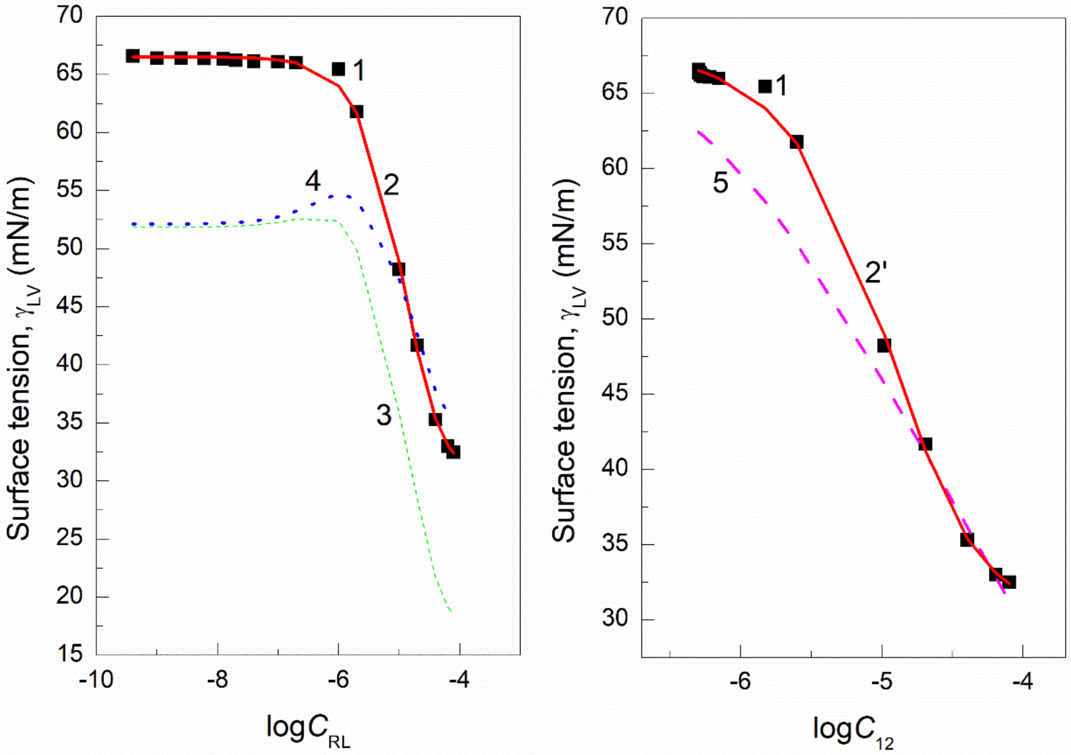

Figure S7. A plot of the surface tension (γ_{LV}) of the aqueous solution of RL and TX165 mixture at the constant TX165 concentration equal to 5 × 10^{-7} mole/dm^3 vs. the logarithm of the RL concentrations (C_{RL}) (a) and the logarithm of the total concentration of the TX165 + RL mixture (C_{12}) (b). Points 1 correspond to the measured values. Curves 2 and 2′ correspond to the values calculated from Eq. (1), curves 3, 4 and 5 correspond to the values calculated from Eqs. (3), (4) and (2), respectively.

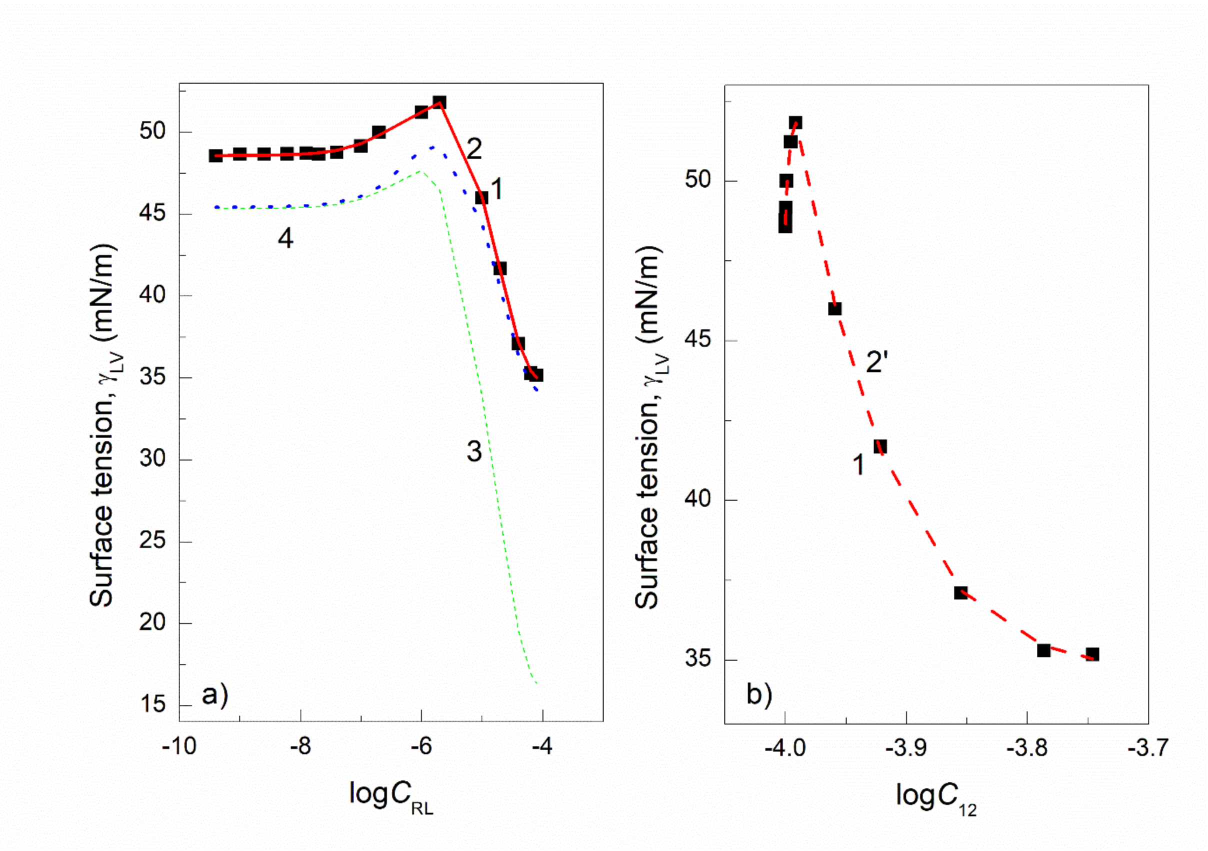

Figure S8. A plot of the surface tension (γ_{LV}) of the aqueous solution of RL and TX165 mixture at the constant TX165 concentration equal to 2 × 10^{-4} mole/dm^3 vs. the logarithm of the RL concentration (C_{RL}) (a) and the logarithm of the total concentration of the TX165 + RL mixture (C_{12}) (b). Points 1 correspond to the measured values. Curves 2 and 2′ correspond to the values calculated from Eq. (1), curves 3 and 4 correspond to the values calculated from Eqs. (3) and (4), respectively.

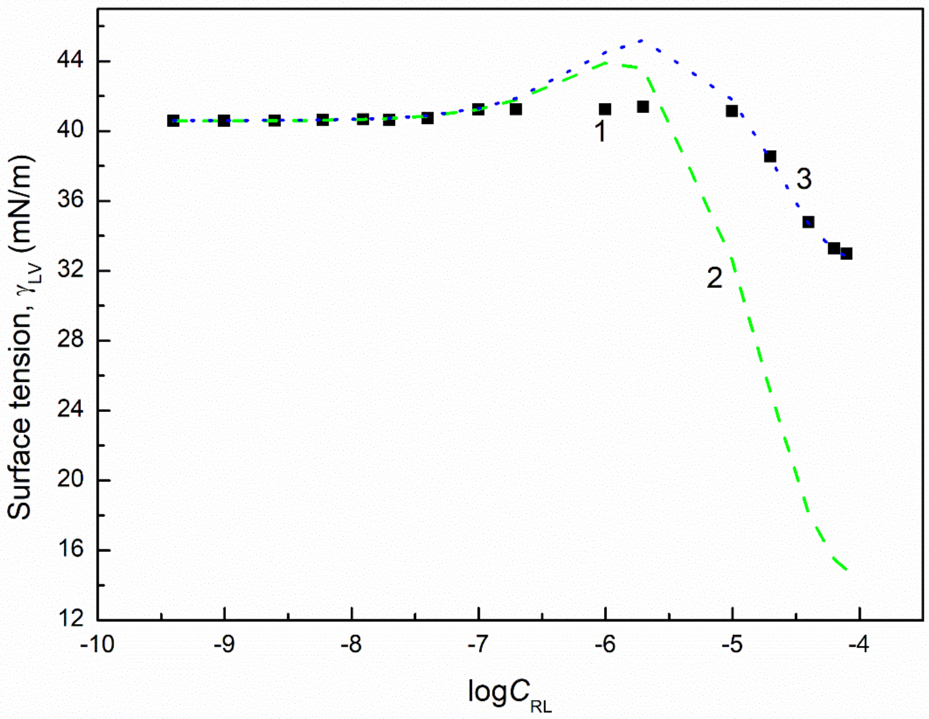

Figure S9. A plot of the surface tension (γ_{LV}) of the aqueous solution of the RL and TX165 mixture at the constant TX165 concentration equal to 1 × 10^{-3} mole/dm^3 vs. the logarithm of the RL concentration (C_{RL}). Points 1 correspond to the measured values. Curves 2 and 3 correspond to the values calculated from Eqs. (3) and (4), respectively.

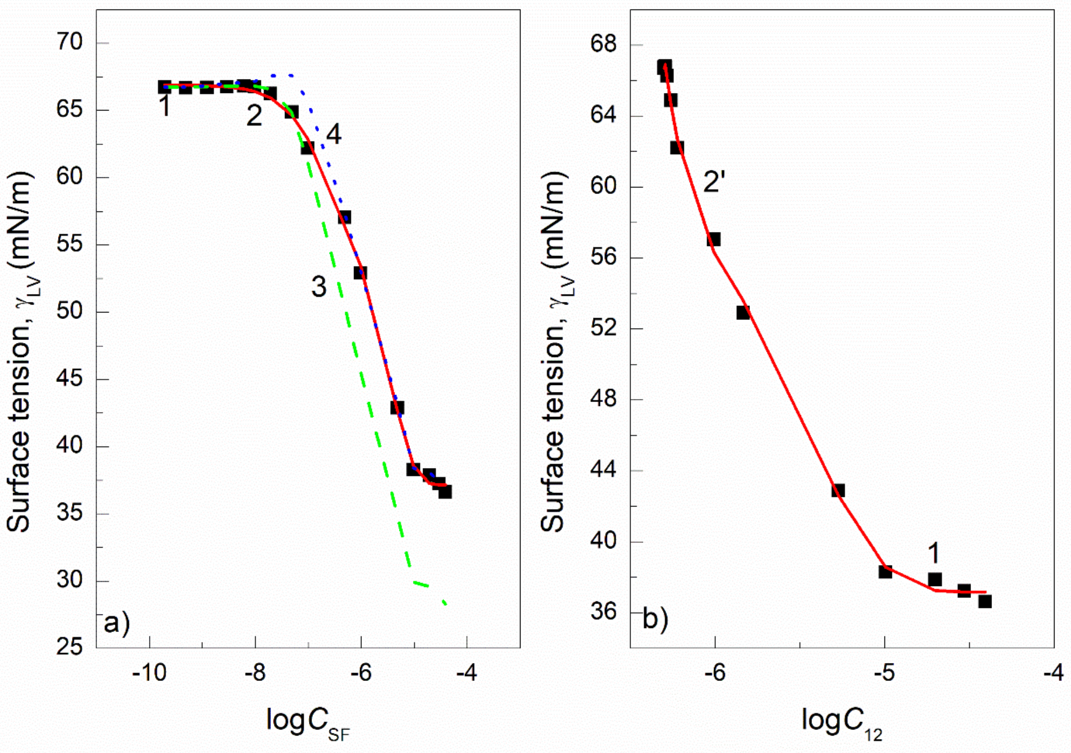

Figure S10. A plot of the surface tension (γ_{LV}) of the aqueous solution of the SF and TX165 mixture at the constant TX165 concentration equal to 5 × 10^{-7} mole/dm^3 vs. the logarithm of the SF concentration (C_{SF}) (a) and the logarithm of the total concentration of the TX165 + SF mixture (C_{12}) (b). Points 1 correspond to the measured values. Curves 2 and 2′ correspond to the values calculated from Eq. (1), curves 3 and 4 correspond to the values calculated from Eqs. (3) and (4), respectively.

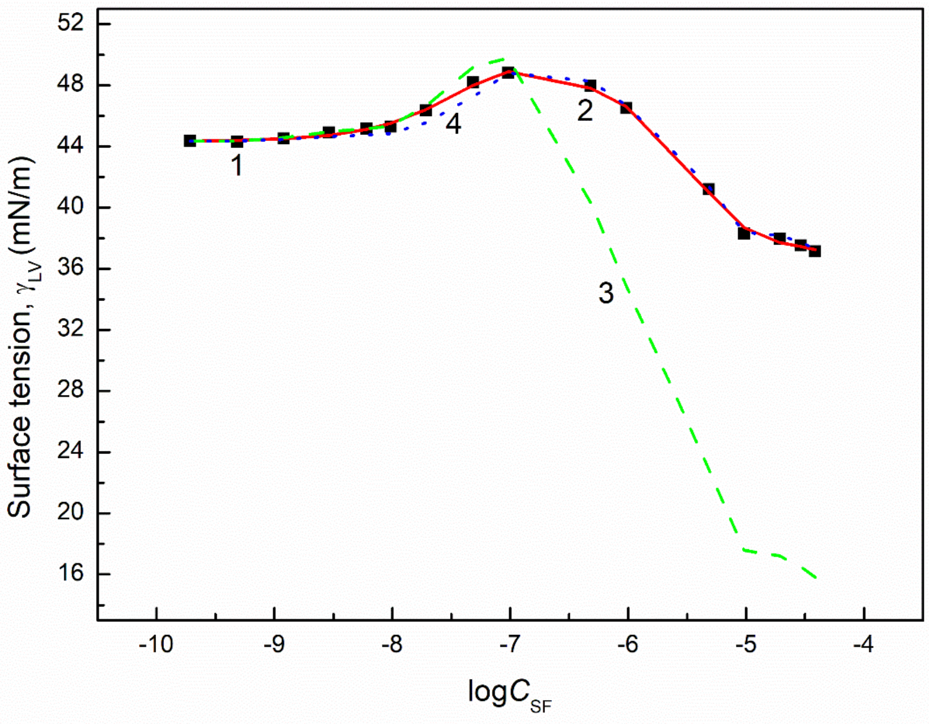

Figure S11. A plot of the surface tension (γ_{LV}) of the aqueous solution of the SF and TX165 mixture at the constant TX165 concentration equal to 2 × 10^{-4} mole/dm^3 vs. the logarithm of the SF concentration (C_{SF}). Points 1 correspond to the measured values. Curves 2, 3 and 4 correspond to the values calculated from Eqs. (1), (3) and (4), respectively.

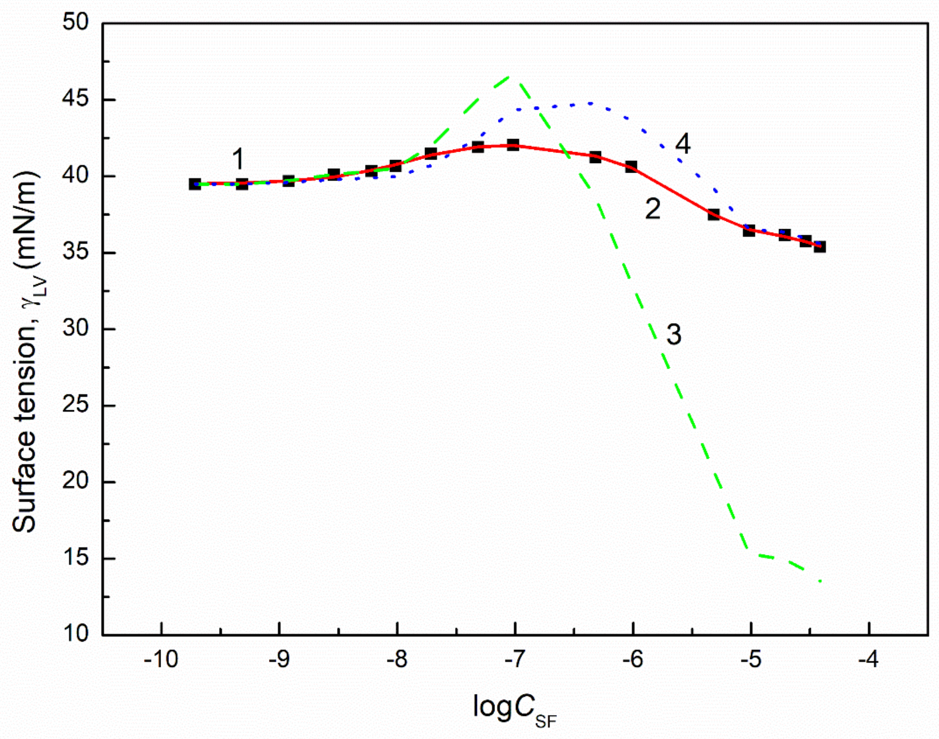

Figure S12. A plot of the surface tension (γ_{LV}) of the aqueous solution of the SF and TX165 mixture at the constant TX165 concentration equal to 1 × 10^{-3} mole/dm^3 vs. the logarithm of the SF concentration (C_{SF}). Points 1 correspond to the measured values. Curves 2, 3 and 4 correspond to the values calculated from Eqs. (1), (3) and (4), respectively.

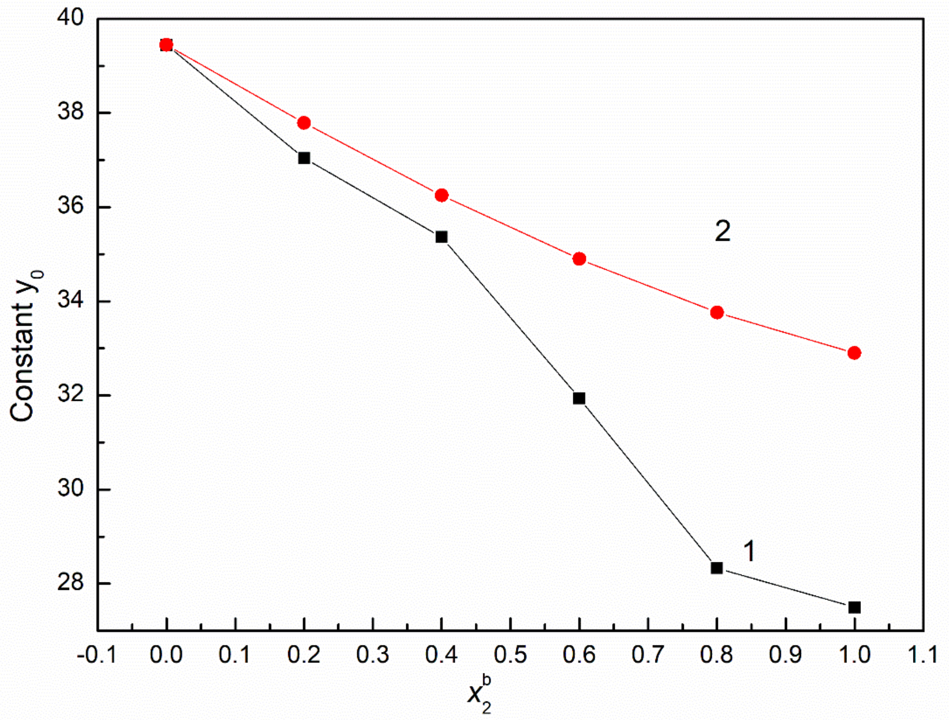

Figure S13. A plot of the constant y_0 in Eq. (1) for the TX165 + RL (curve 1) and TX165 + SF (curve 2) aqueous solutions vs. the biosurfactant mole fraction in the mixture in the bulk phase (x_2^b).

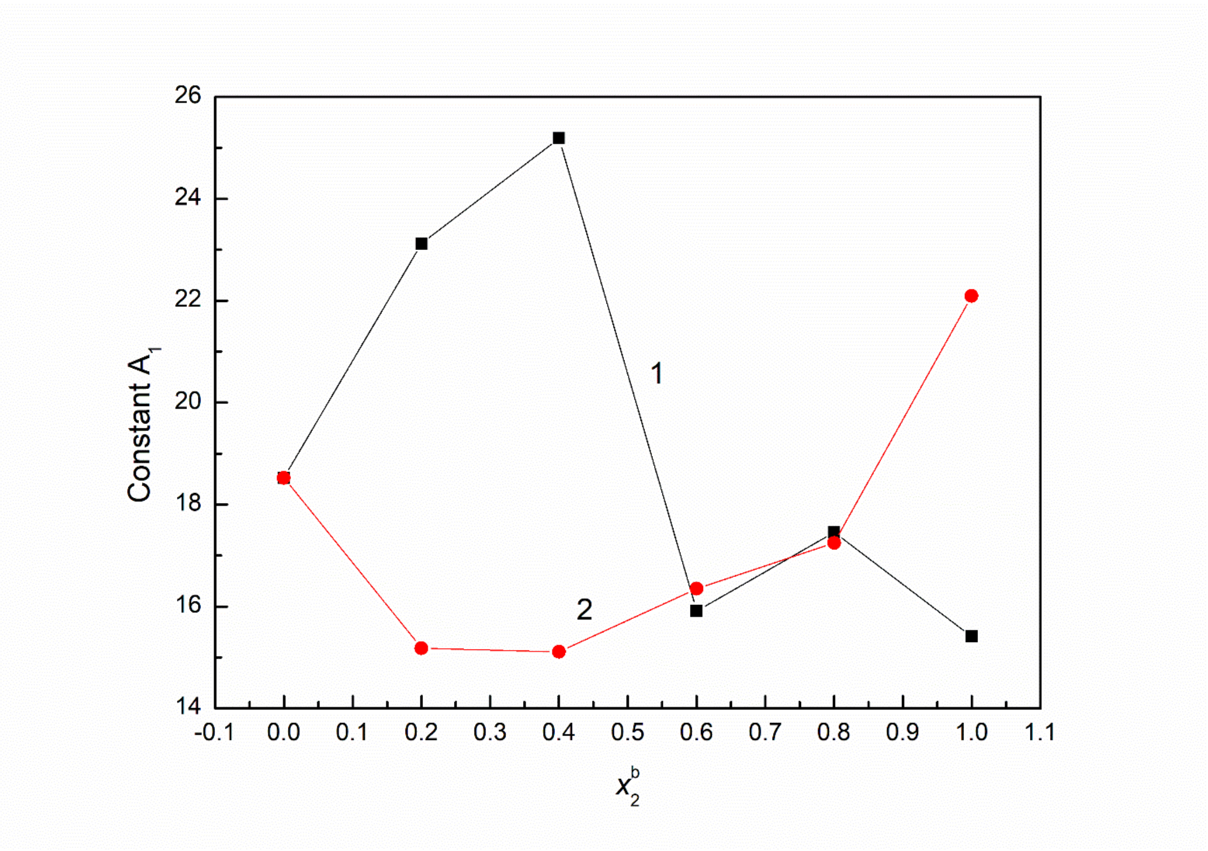

Figure S14. A plot of the constant A_1 in Eq. (1) for the TX165 + RL (curve 1) and TX165 + SF (curve 2) aqueous solutions vs. the biosurfactant mole fraction in the mixture in the bulk phase (x_2^b).

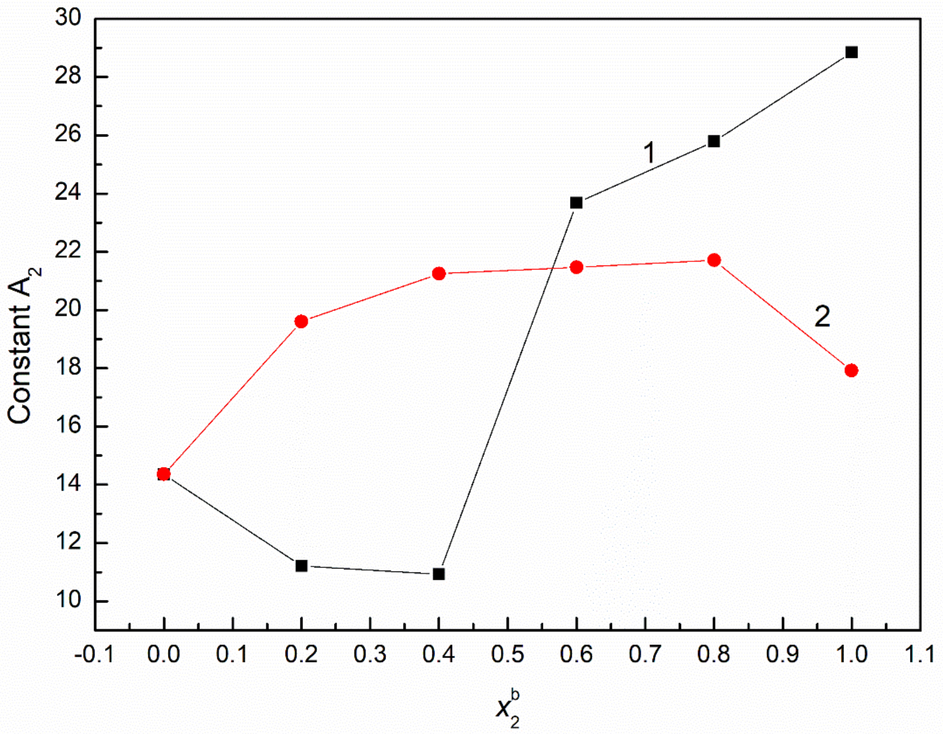

Figure S15. A plot of the constant A_2 in Eq. (1) for the TX165 + RL (curve 1) and TX165 + SF (curve 2) aqueous solutions vs. the biosurfactant mole fraction in the mixture in the bulk phase (x_2^b).

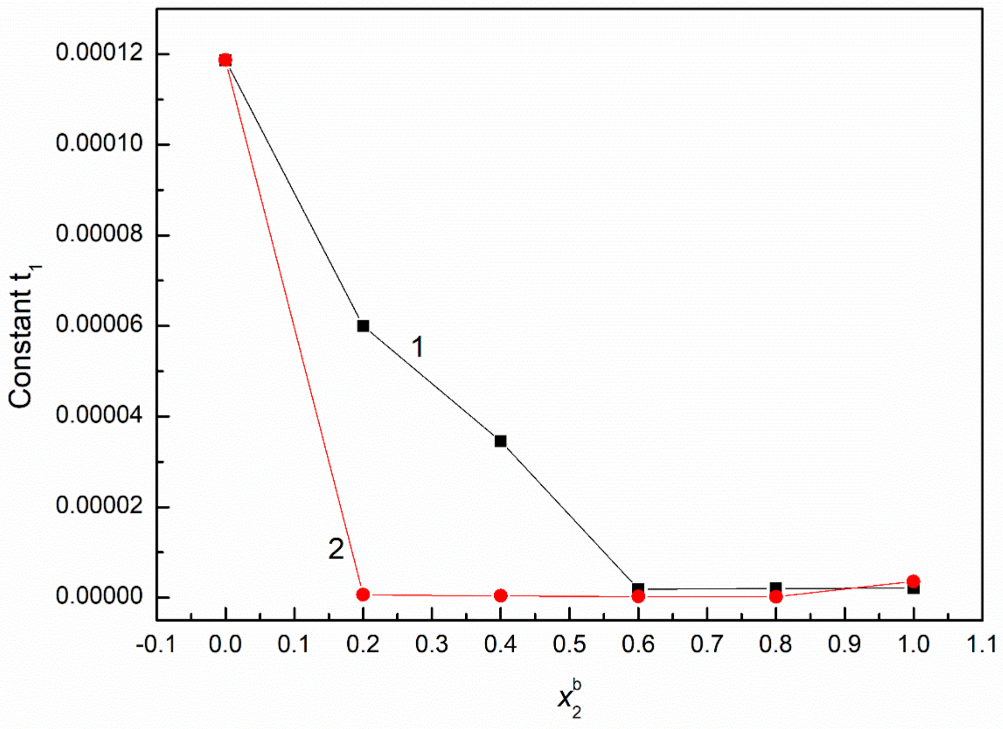

Figure S16. A plot of the constant t_1 in Eq. (1) for the TX165 + RL (curve 1) and TX165 + SF (curve 2) aqueous solutions vs. the biosurfactant mole fraction in the mixture in the bulk phase (x_2^b).

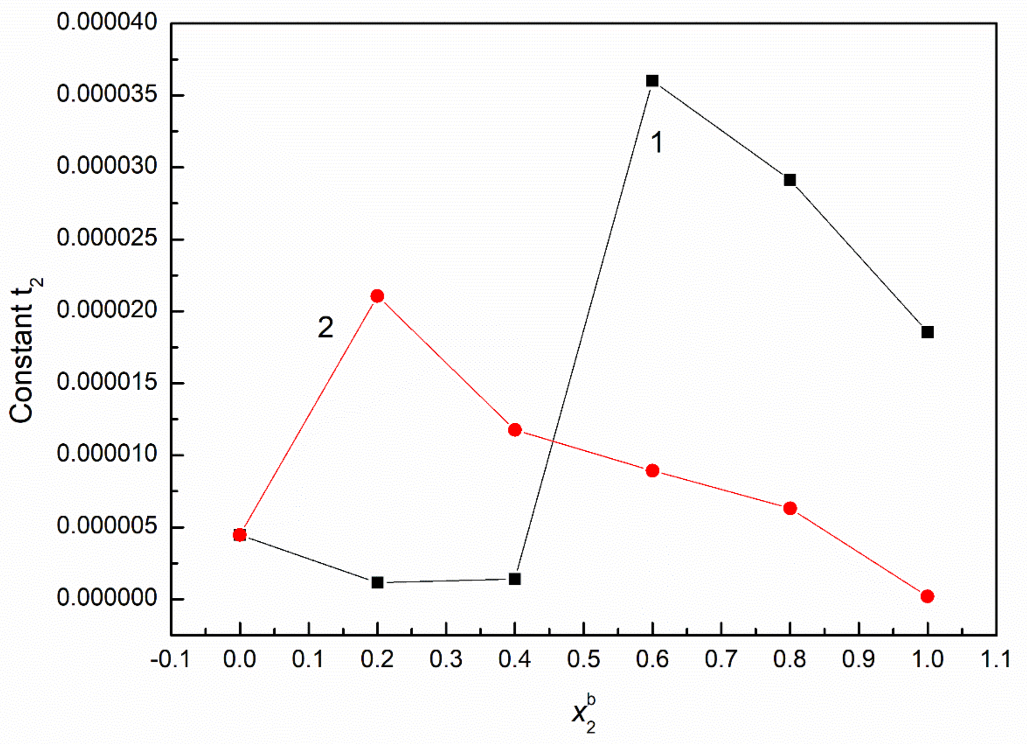

Figure S17. A plot of the constant t_2 in Eq. (1) for the TX165 + RL (curve 1) and TX165 + SF (curve 2) aqueous solutions vs. the biosurfactant mole fraction in the mixture in the bulk phase (x_2^b).

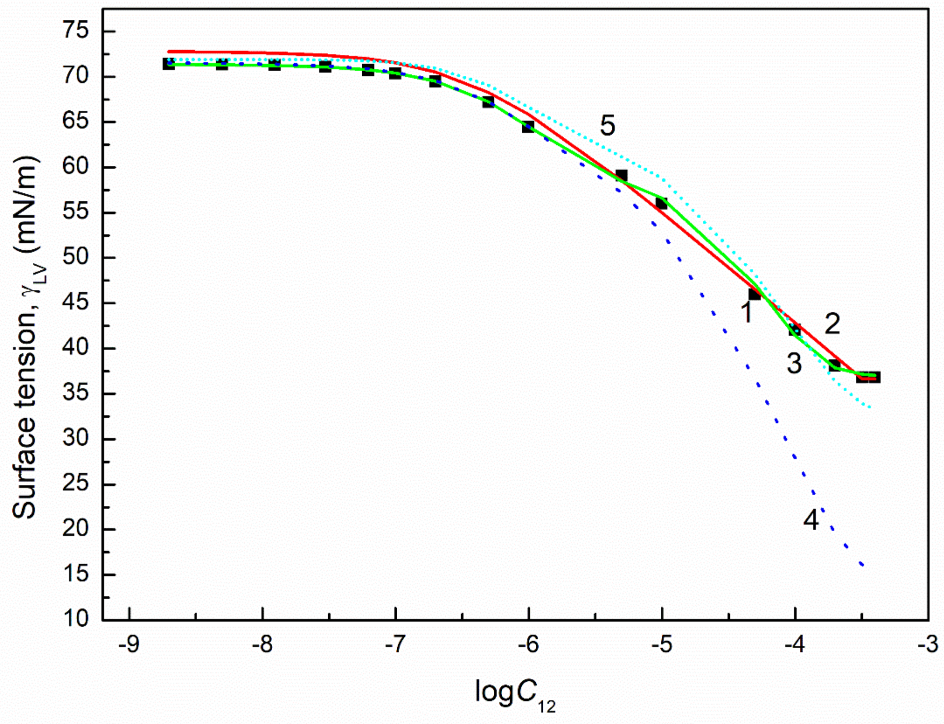

Figure S18. A plot of the surface tension (γ_{LV}) of the aqueous solution of the RL and TX165 mixture at the RL mole fraction equal to 0.2 vs. the logarithm of the total concentration of the TX165 + RL mixture (C_{12}). Points 1 correspond to the measured values. Curves 2, 3, 4 and 5 correspond to the values calculated from Eqs. (2), (1), (3) and (4), respectively.

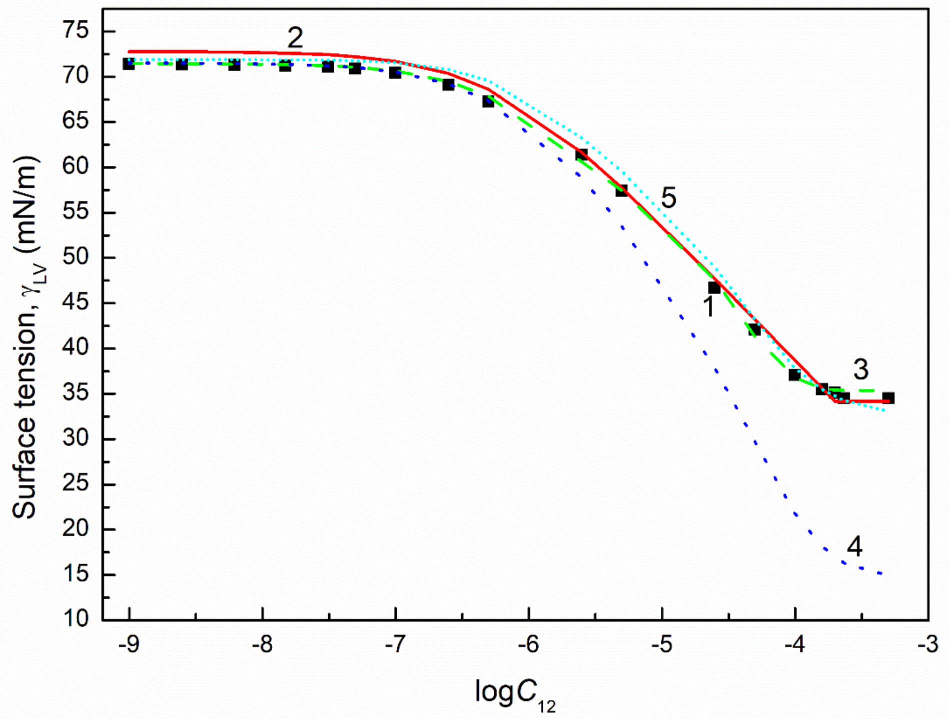

Figure S19. A plot of the surface tension (γ_{LV}) of the aqueous solution of the RL and TX165 mixture at the RL mole fraction equal to 0.4 vs. the logarithm of the total concentration of the TX165 + RL mixture (C_{12}). Points 1 correspond to the measured values. Curves 2, 3, 4 and 5 correspond to the values calculated from Eqs. (2), (1), (3) and (4), respectively.

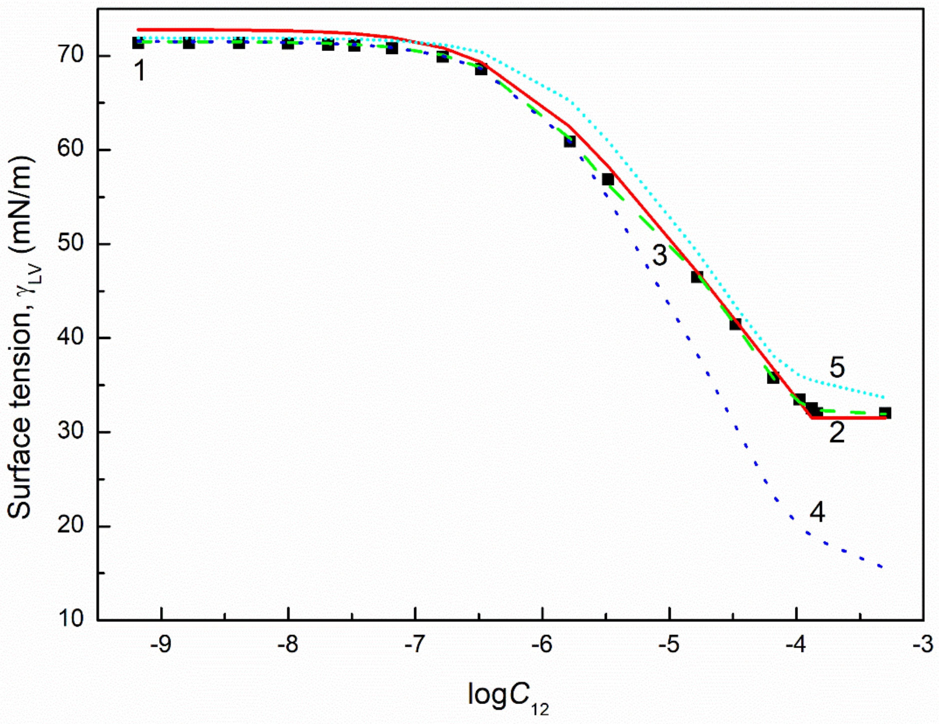

Figure S20. A plot of the surface tension (γ_{LV}) of the aqueous solution of the RL and TX165 mixture at the RL mole fraction equal to 0.6 vs. the logarithm of the total concentration of the TX165 + RL mixture (C_{12}). Points 1 correspond to the measured values. Curves 2, 3, 4 and 5 correspond to the values calculated from Eqs. (2), (1), (3) and (4), respectively.

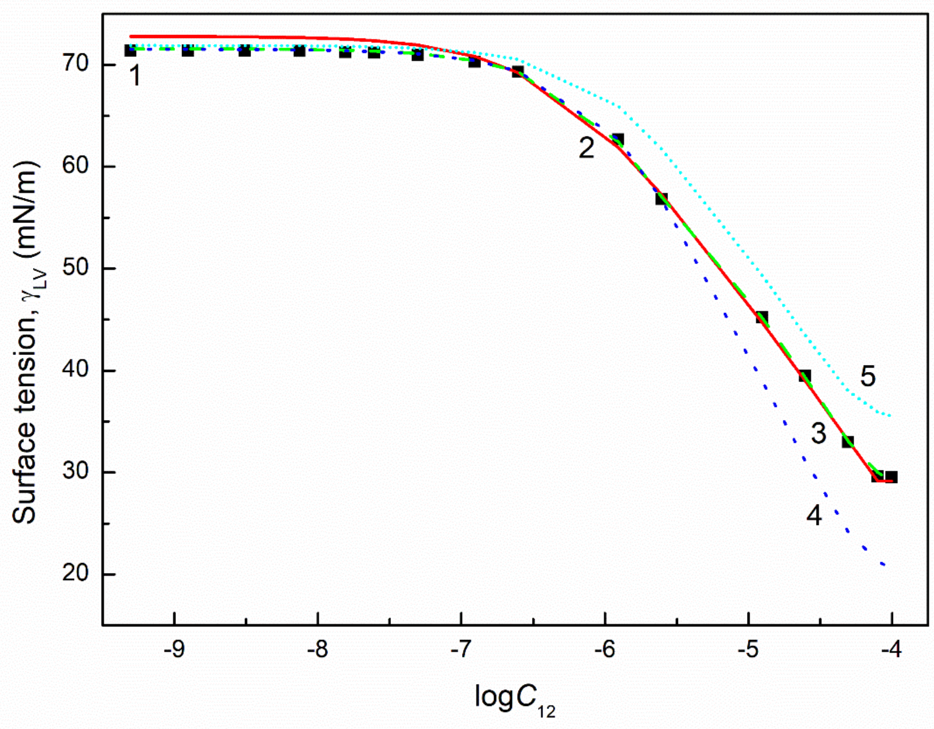

Figure S21. A plot of the surface tension (γ_{LV}) of the aqueous solution of the RL and TX165 mixture at the RL mole fraction equal to 0.8 vs. the logarithm of the total concentration of the TX165 + RL mixture (C_{12}). Points 1 correspond to the measured values. Curves 2, 3, 4 and 5 correspond to the values calculated from Eqs. (2), (1), (3) and (4), respectively.

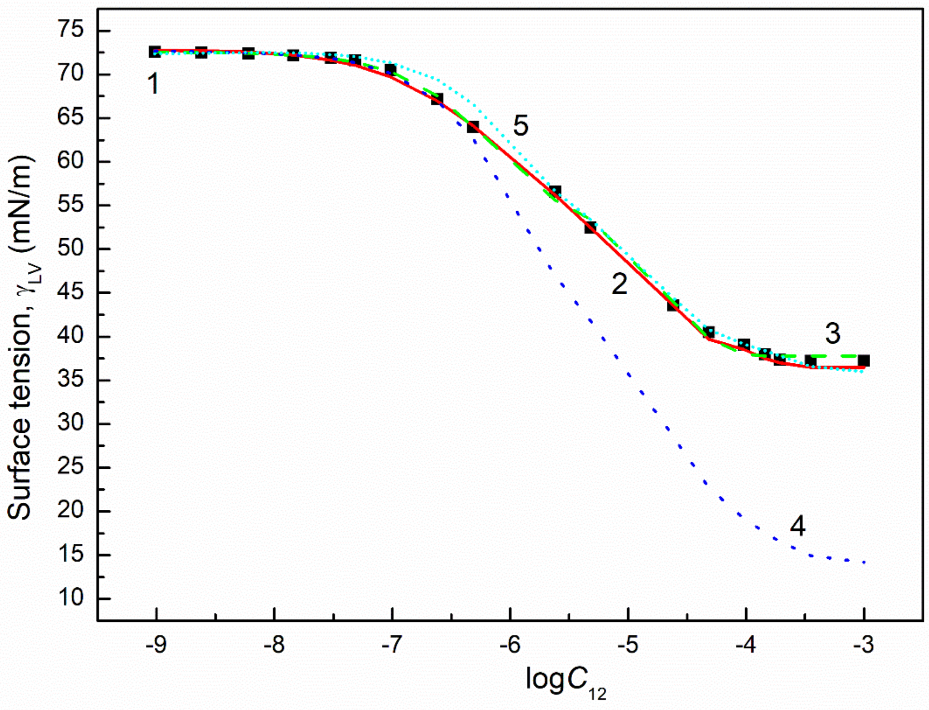

Figure S22. A plot of the surface tension (γ_{LV}) of the aqueous solution of the SF and TX165 mixture at the SF mole fraction equal to 0.2 vs. the logarithm of the total concentration of the TX165 + SF mixture (C_{12}). Points 1 correspond the measured values. Curves 2, 3, 4 and 5 correspond to the values calculated from Eqs. (2), (1), (3) and (4), respectively.

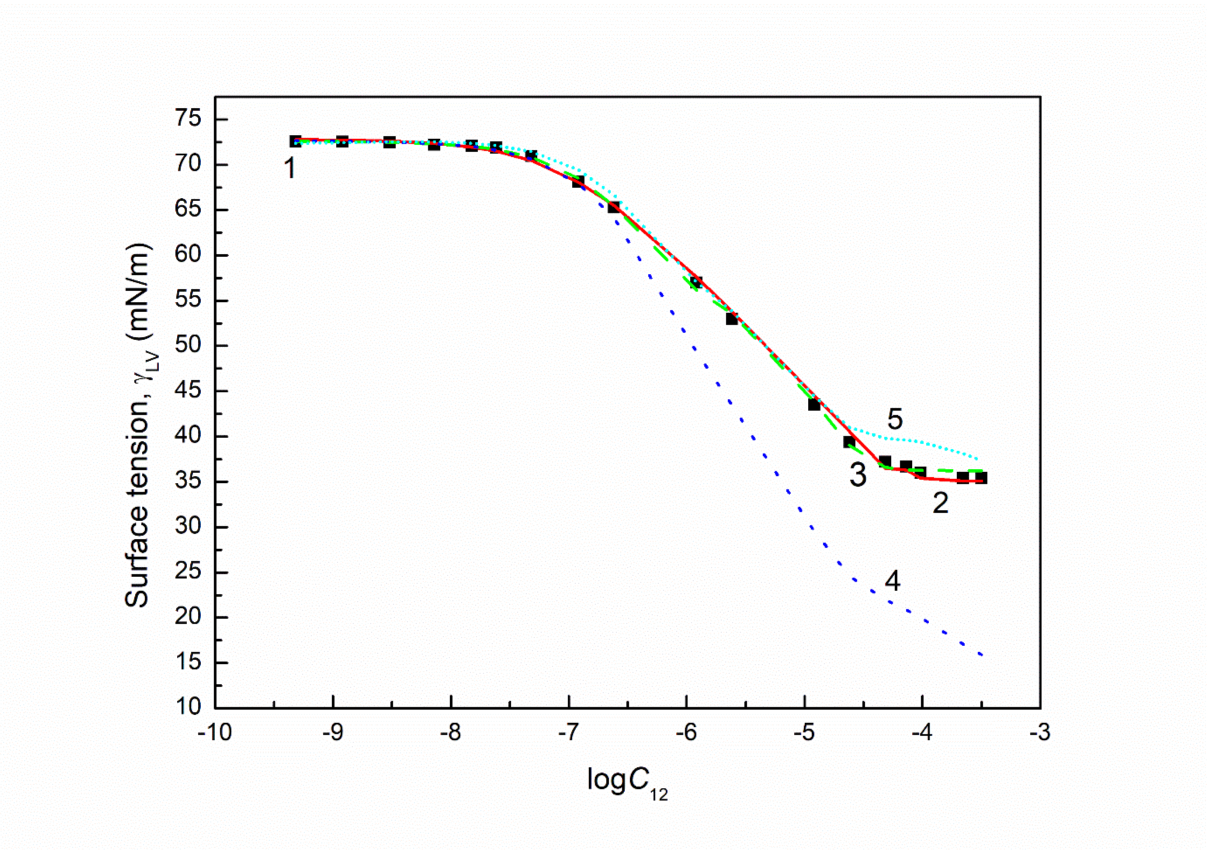

Figure S23. A plot of the surface tension (γ_{LV}) of the aqueous solution of the SF and TX165 mixture at the SF mole fraction equal to 0.4 vs. the logarithm of the total concentration of the TX165 + SF mixture (C_{12}). Points 1 correspond to the measured values. Curves 2, 3, 4 and 5 correspond to the values calculated from Eqs. (2), (1), (3) and (4), respectively.

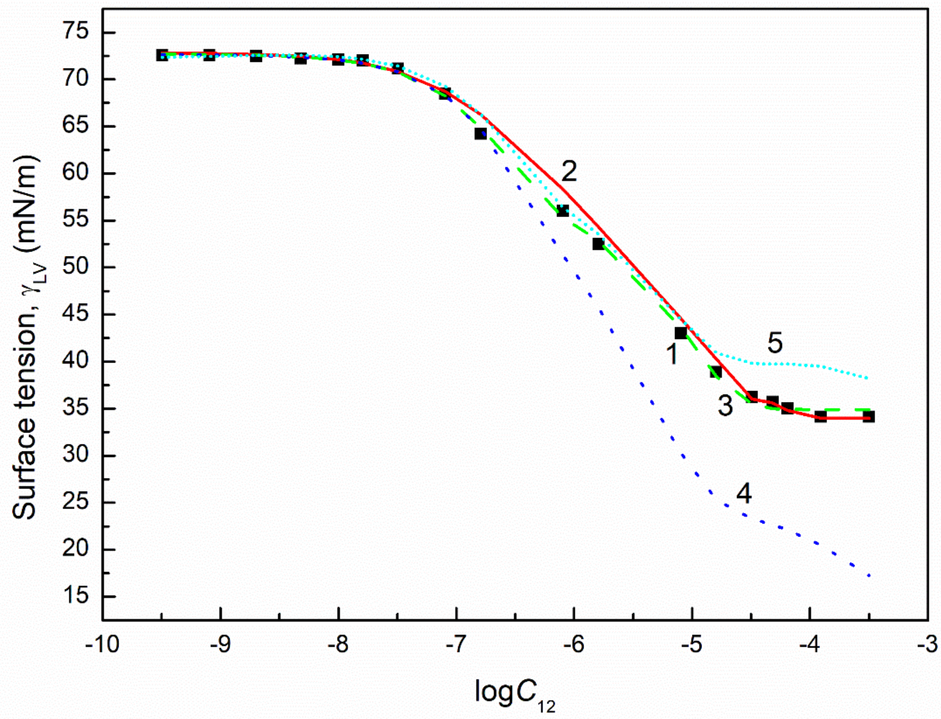

Figure S24. A plot of the surface tension (γ_{LV}) of the aqueous solution of the SF and TX165 mixture at the SF mole fraction equal to 0.6 vs. the logarithm of the total concentration of the TX165 + SF mixture (C_{12}). Points 1 correspond to the measured values. Curves 2, 3, 4 and 5 correspond to the values calculated from Eqs. (2), (1), (3) and (4), respectively.

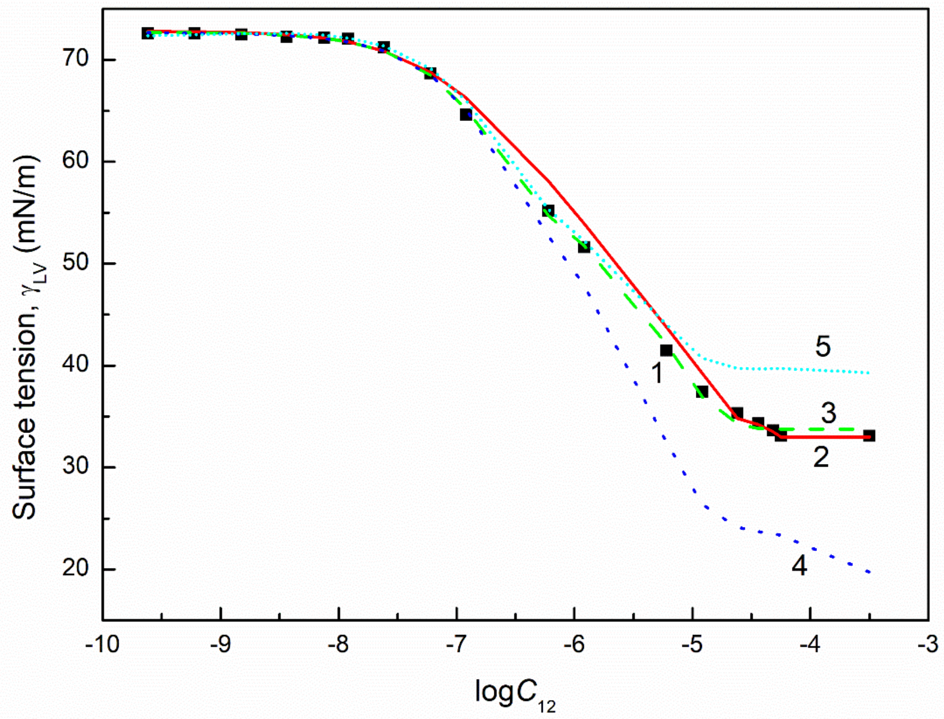

Figure S25. A plot of the surface tension (γ_{LV}) of the aqueous solution of the SF and TX165 mixture at the SF mole fraction equal to 0.8 vs. the logarithm of the total concentration of TX165 + SF mixture (C_{12}). Points 1 correspond to the measured values. Curves 2, 3, 4 and 5 correspond to the values calculated from Eqs. (2), (1), (3) and (4), respectively.

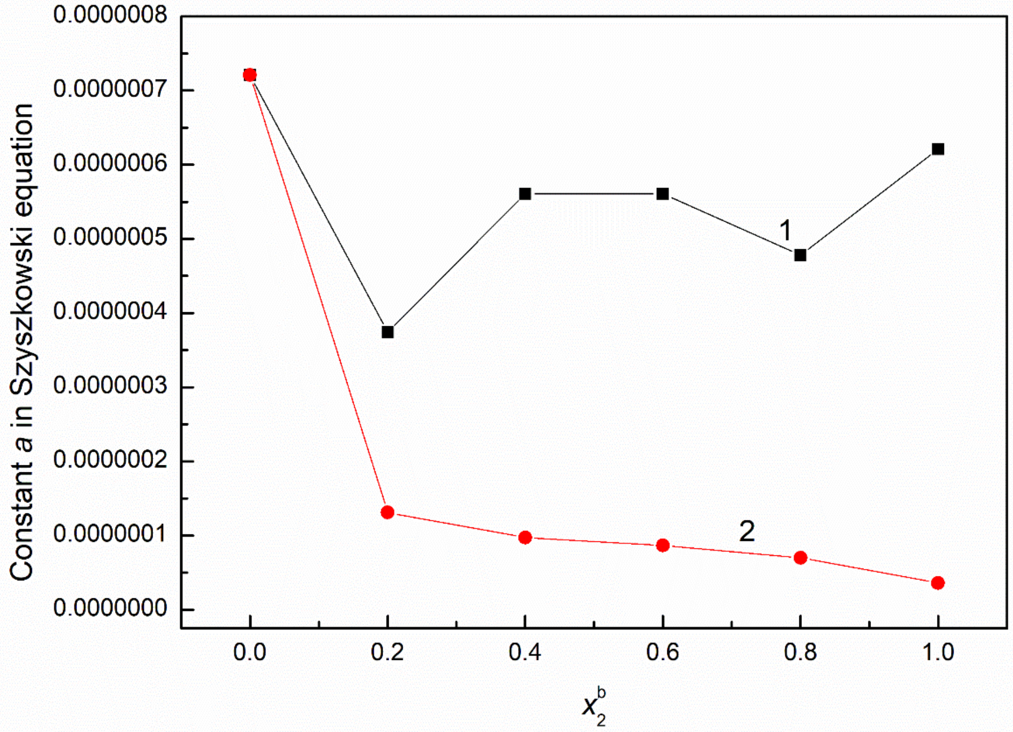

Figure S26. A plot of the constant a in the Szyszkowski equation (Eq. (2)) for the TX165 + RL (curve 1) and TX165 + SF (curve 2) aqueous solutions vs. the biosurfactant mole fraction in the mixture in the bulk phase (x_2^b).

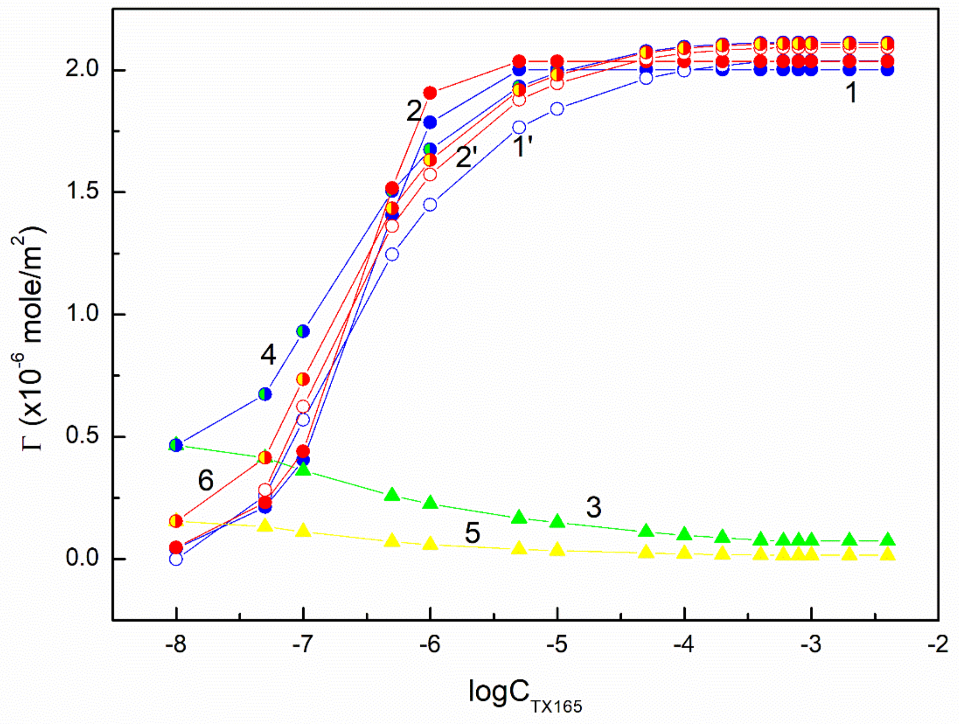

Figure S27. A plot of the surface concentration (Γ) of TX165 (curves 1, 1′, 2, 2′), RL (curve 3) and SF (curve 5) vs. the logarithm of TX165 concentration (C_{TX165}) at the constant biosurfactant concentration equal to 0.00625 mg/dm^3. Curves 1 and 2 correspond to the values calculated from Eq. (5), curves 1′, 2′ 3, 5 to the values calculated from Eq. (6). Curves 4 and 6 correspond to the sum values calculated from Eq. (6) for the TX165 + RL and TX165 + SF mixture, respectively.

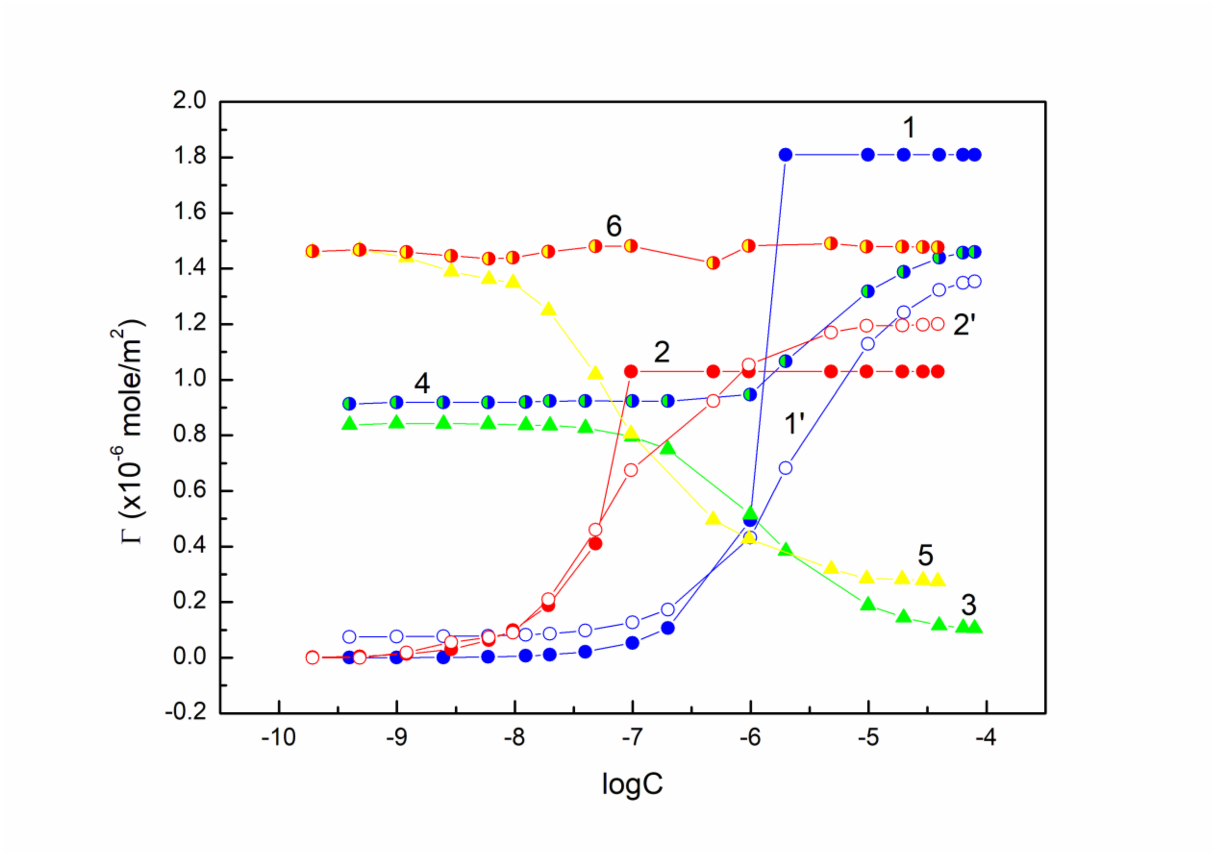

Figure S28. A plot of the surface concentration (Γ) of RL (curves 1, 1′), SF (curves 2, 2′) and TX165 (curves 3 and 5) vs. the logarithm of biosurfactant concentration (C) at the constant TX165 concentration equal to 5×10^{-7} mole/dm^3. Curves 1 and 2 correspond to the values calculated from Eq. (5), curves 1′, 2′ 3, 5 to the values calculated from Eq. (6). Curves 4 and 6 correspond to the sum values calculated from Eq. (6) for the TX165 + RL and TX165 + SF mixture, respectively.

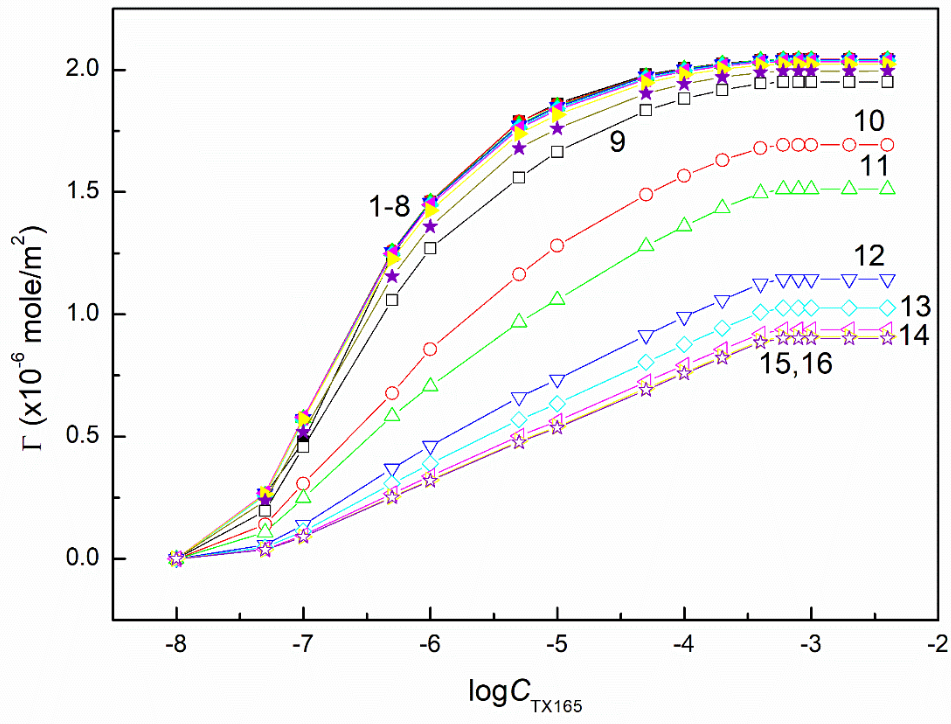

Figure S29. A plot of the surface concentration (*Γ*) of TX165 calculated from Eq. (6) in the TX165 + RL mixture vs. the logarithm of its concentration (C_{TX165}). Curves 1 – 16 correspond to the constant RL concentration equal to 0.0002, 0.0005, 0.00125, 0.003, 0.00625, 0.01, 0.02, 0.05, 0.1, 0.5, 1, 5, 10, 20, 30 and 40 mg/dm^3, respectively.

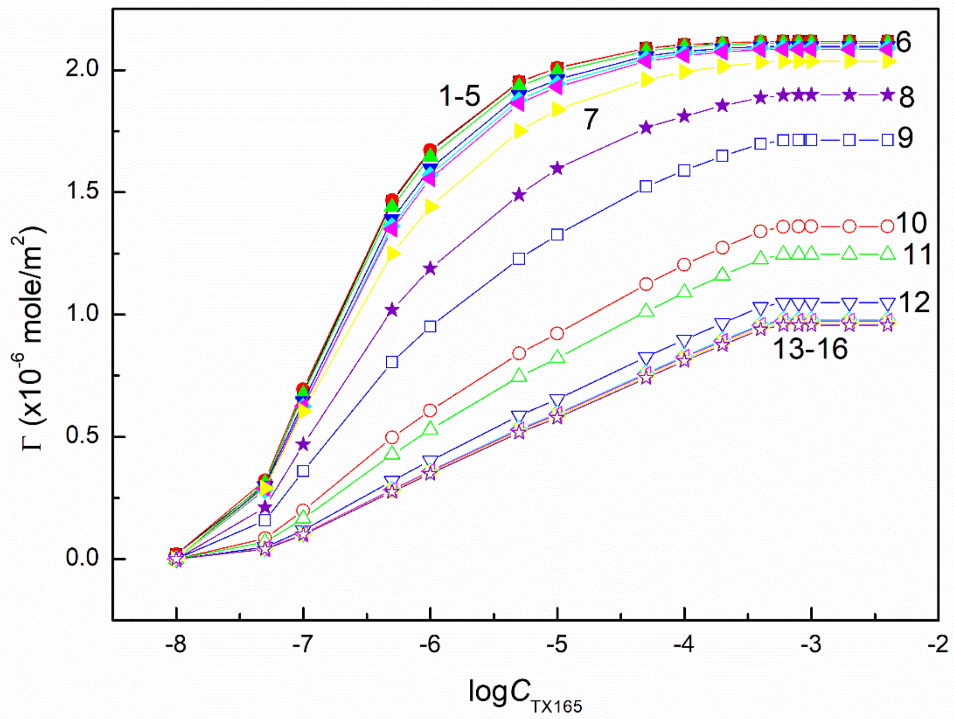

Figure S30. A plot of the surface concentration (Γ) of TX165 calculated from Eq. (6) in the TX165 + SF mixture vs. the logarithm of its concentration (C_{TX165}). Curves 1 – 16 correspond to the constant SF concentration equal to 0.0002, 0.0005, 0.00125, 0.003, 0.00625, 0.01, 0.02, 0.05, 0.1, 0.5, 1, 5, 10, 20, 30 and 40 mg/dm^3, respectively.

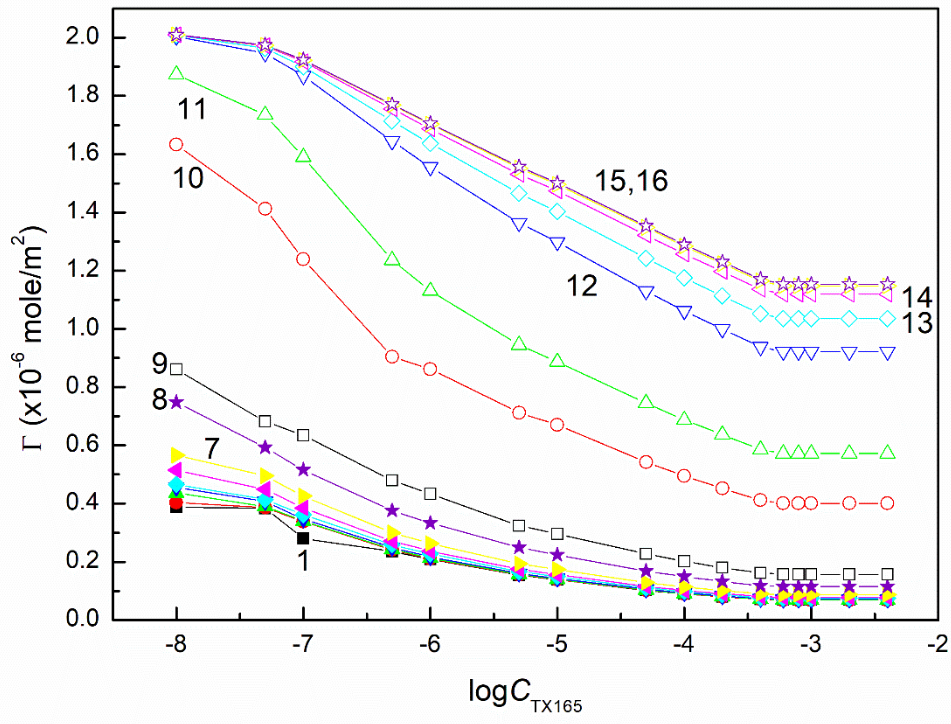

Figure S31. A plot of the surface concentration (Γ) of RL calculated from Eq. (6) in the TX165 + RL mixture vs. the logarithm of TX165 concentration (C_{TX165}). Curves 1 – 16 correspond to the constant RL concentration equal to 0.0002, 0.0005, 0.00125, 0.003, 0.00625, 0.01, 0.02, 0.05, 0.1, 0.5, 1, 5, 10, 20, 30 and 40 mg/dm^3, respectively.

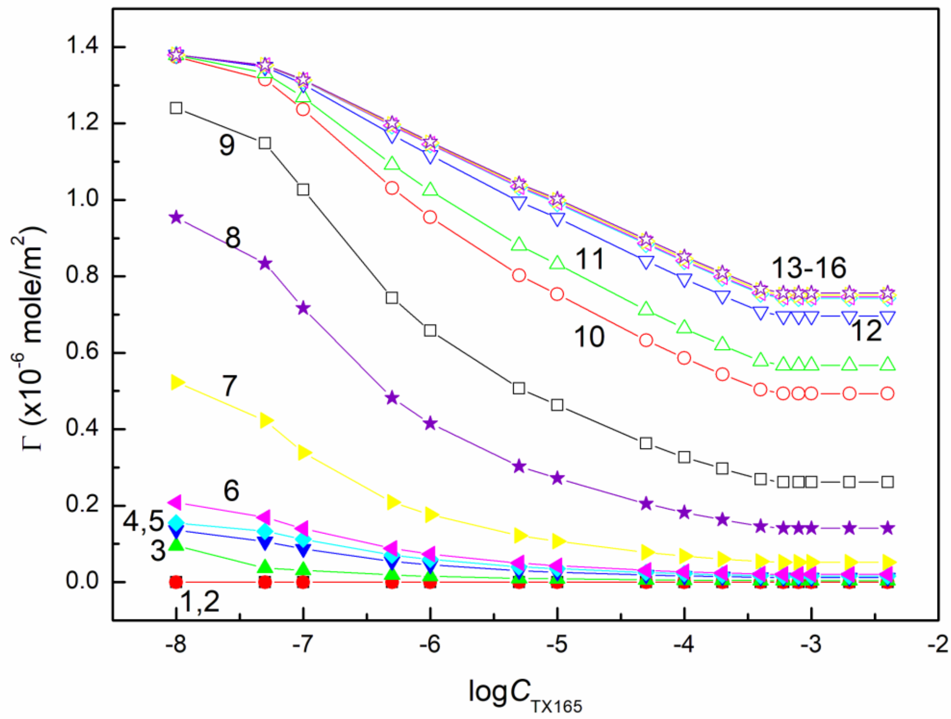

Figure S32. A plot of the surface concentration (Γ) of SF calculated from Eq. (6) in the TX165 + SF mixture vs. the logarithm of TX165 concentration (C_{TX165}). Curves 1 – 16 correspond to the constant SF concentration equal to 0.0002, 0.0005, 0.00125, 0.003, 0.00625, 0.01, 0.02, 0.05, 0.1, 0.5, 1, 5, 10, 20, 30 and 40 mg/dm^3, respectively.

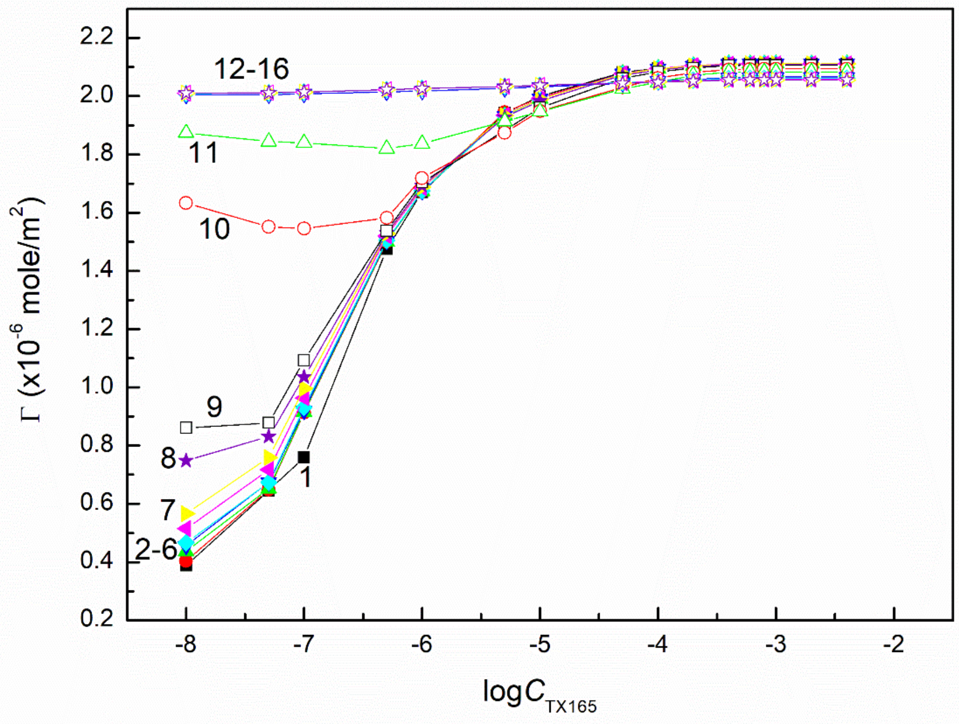

Figure S33. A plot of the total surface concentration (Γ) of the TX165 + RL mixture calculated from Eq. (6) vs. the logarithm of TX165 concentration (C_{TX165}). Curves 1 – 16 correspond to the constant RL concentration equal to 0.0002, 0.0005, 0.00125, 0.003, 0.00625, 0.01, 0.02, 0.05, 0.1, 0.5, 1, 5, 10, 20, 30 and 40 mg/dm^3, respectively.

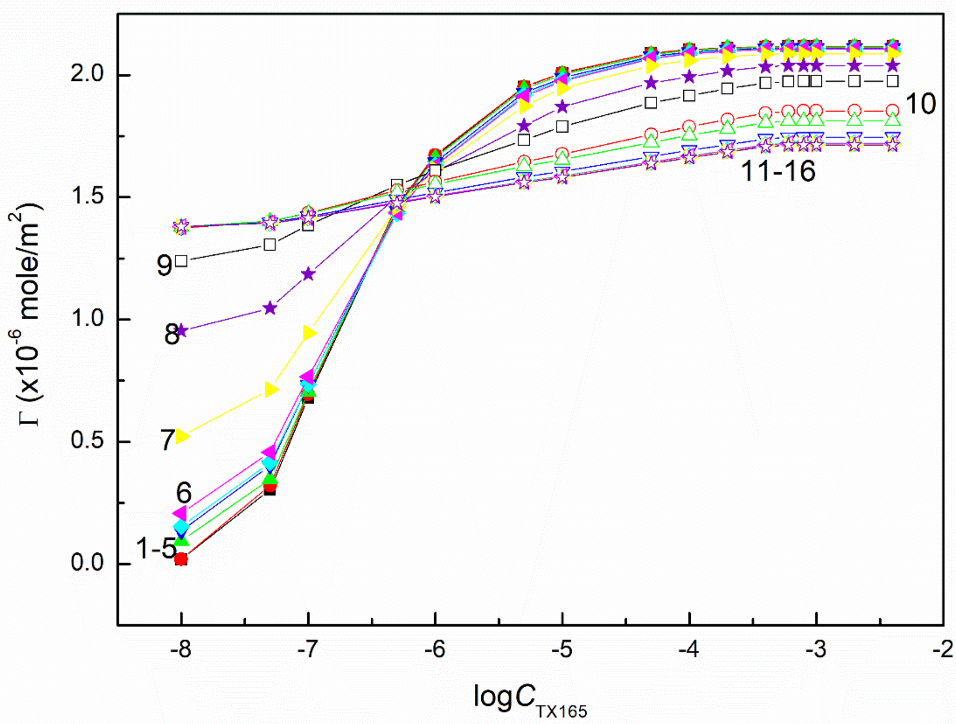

Figure S34. A plot of the total surface concentration (Γ) of the TX165 + SF mixture calculated from Eq. (6) vs. the logarithm of TX165 concentration (C_{TX165}). Curves 1 – 16 correspond to the constant SF concentration equal to 0.0002, 0.0005, 0.00125, 0.003, 0.00625, 0.01, 0.02, 0.05, 0.1, 0.5, 1, 5, 10, 20, 30 and 40 mg/dm^3, respectively.

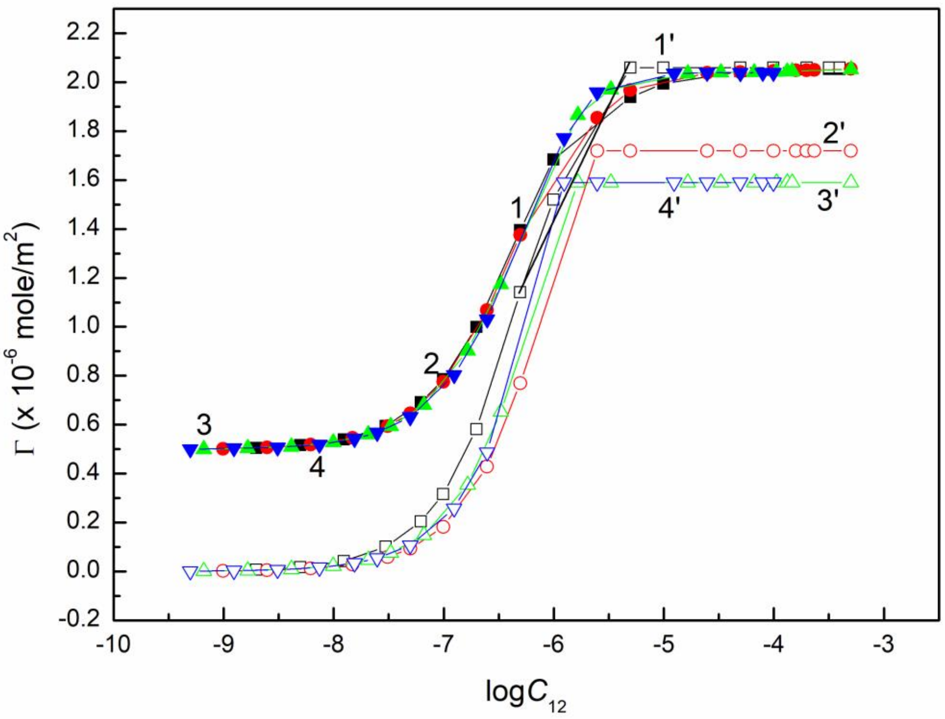

Figure S35. A plot of the total surface concentration (Γ) of the TX165 + RL mixture calculated from Eq. (6) (curves 1, 2, 3 and 4) and Gibbs surface concentration calculated from Eq. (5) vs. the logarithm of the total concentration of RL+TX165 mixture (C_{12}). Curves 1 and 1′ correspond to the RL mole fraction in the mixture in the bulk phase equal to 0.2, curves 2 and 2′ to 0.4, curves 3 and 3′ to 0.6 and curves 4 and 4′ correspond to the RL mole fraction in the mixture in the bulk phase equal 0.8, respectively.

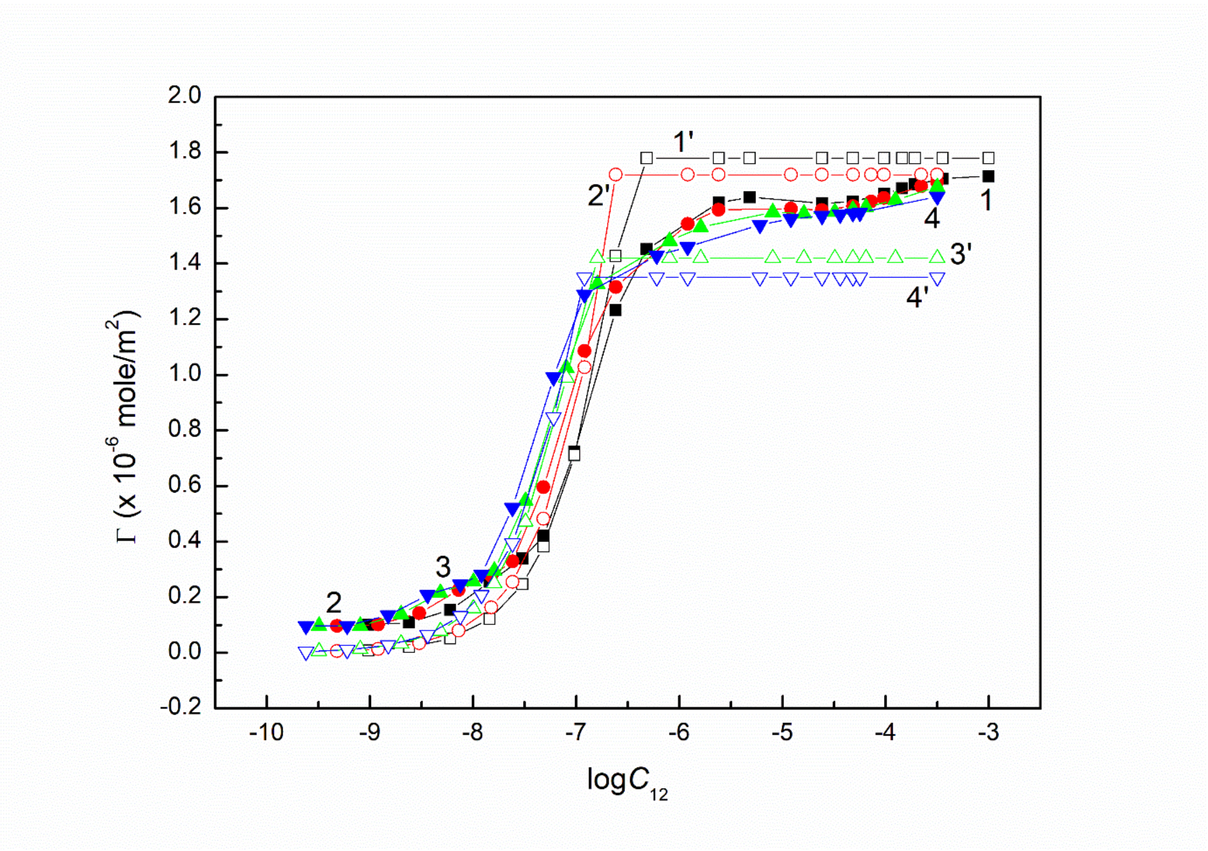

Figure S36. A plot of the total surface concentration (Γ) of the TX165 + SF mixture calculated from Eq. (6) (curves 1, 2, 3 and 4) and Gibbs surface concentration calculated from Eq. (5) vs. the logarithm of the total concentration of TX165 + SF mixture (C_{12}). Curves 1 and 1′ correspond to the SF mole fraction in the mixture in the bulk phase equal to 0.2, curves 2 and 2′ to 0.4, curves 3 and 3′ to 0.6 and curves 4 and 4′ correspond to the SF mole fraction in the mixture in the bulk phase equal 0.8, respectively.

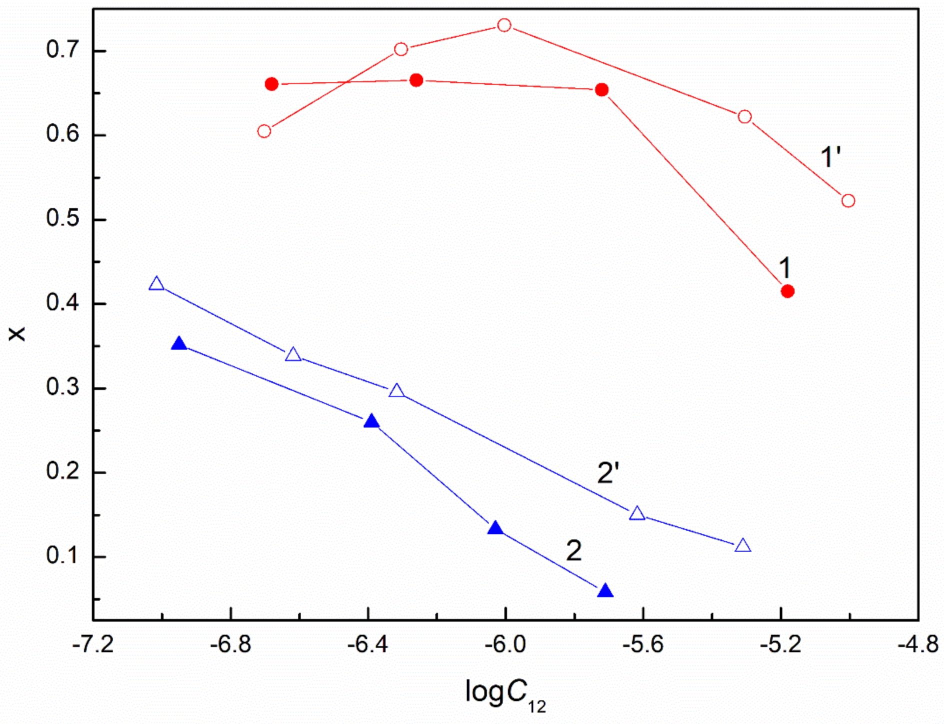

Figure S37. A plot of the TX165 mole fraction in the mixture with RL (curves 1 and 1′) and SF (curves 2 and 2′) (x) at the constant biosurfactant mole fraction in the mixture in the bulk phase equal to 0.2 vs. the total concentration of the TX165 + RL mixture (C_{12}). Curves 1 and 2 correspond to the values obtained from Eq. (7), curves 1′ and 2′ correspond to the values calculated from the expression $x_1^S = \frac{\pi_1}{\pi_1+\pi_2}$

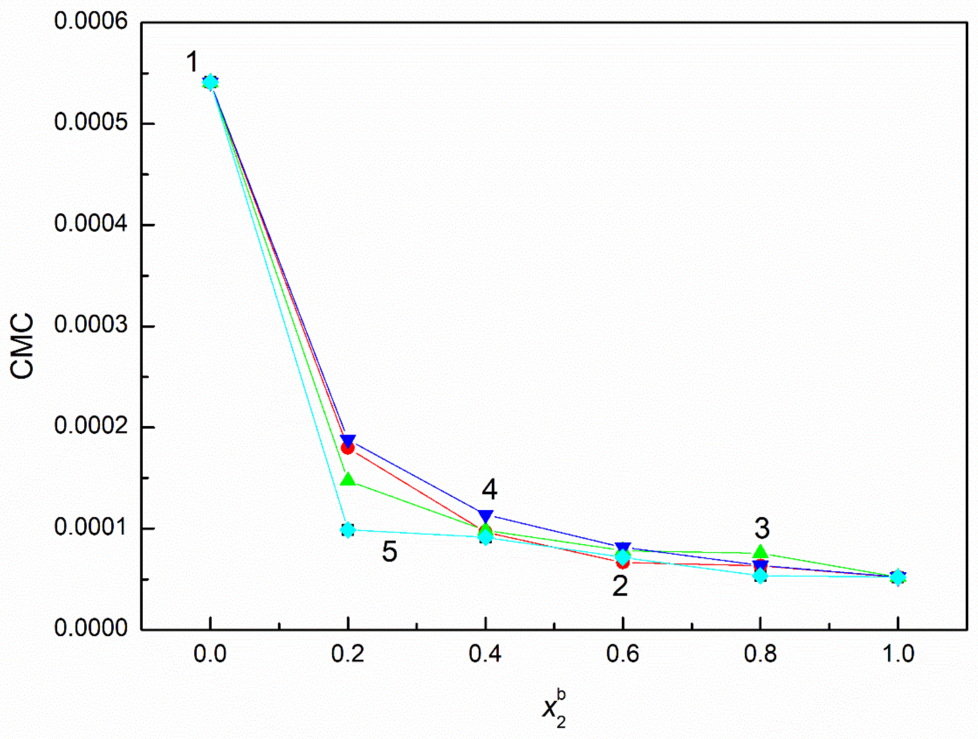

Figure S38. A plot of the CMC values of TX165 + RL and their mixtures vs. the RL mole fraction in the mixture in the bulk phase (x_2^b). Curve 1 corresponds to the CMC values of mixtures determined from surface tension isotherms, curves 2 and 3 correspond to the CMC calculated from Eq. (10), curves 4 and 5 to the values calculated from Eqs. (9) and (15).

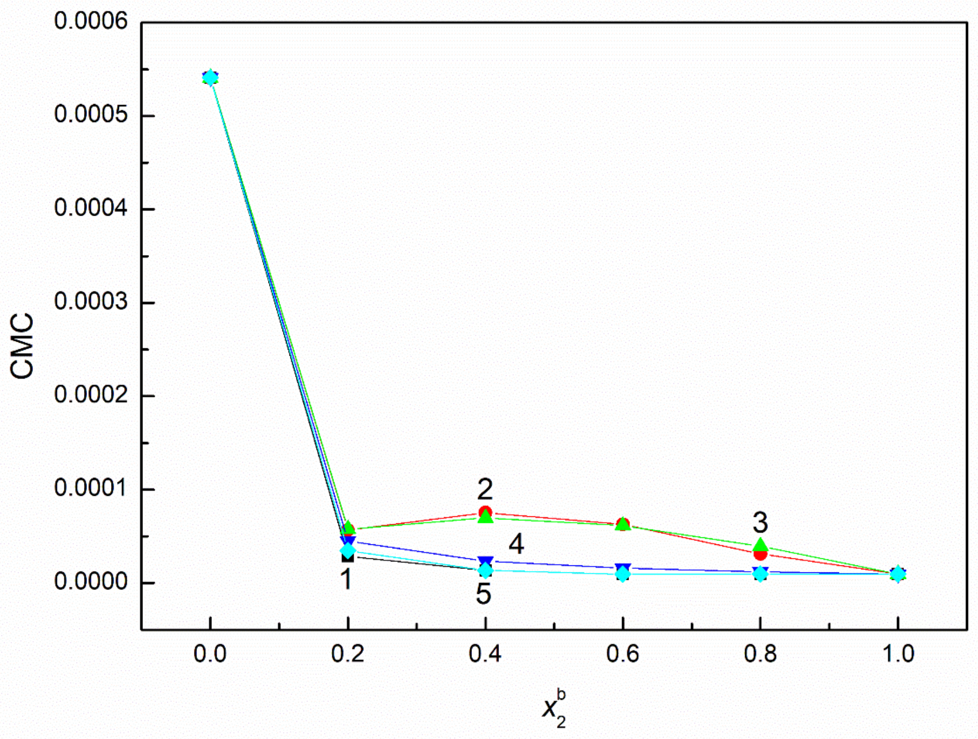

Figure S39. A plot of the CMC values of TX165 + SF and their mixtures vs. the SF mole fraction in the mixture in the bulk phase (x_2^b). Curve 1 corresponds to the CMC values of mixtures determined from surface tension isotherms, curves 2 and 3 correspond to the CMC calculated from Eq. (10), curves 4 and 5 to the values calculated from Eqs. (9) and (15).

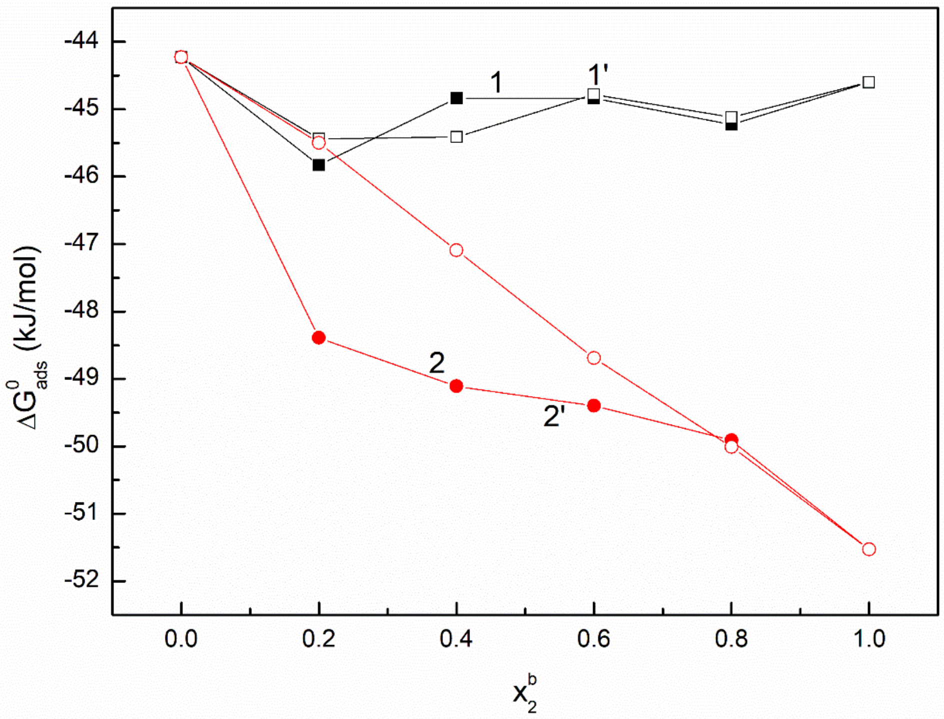

Figure S40. A plot of the Gibbs standard free energy of TX165 + RL (curves 1 and 1′) and TX165 + SF (curves 2 and 2′) adsorption at the water-air interface vs. the biosurfactant mole fraction in the mixture in the bulk phase (x_2^b). Curves 1 and 2 correspond to values calculated from Eq. (16), curves 1′ and 2′ to values calculated from Eq. (17), respectively.

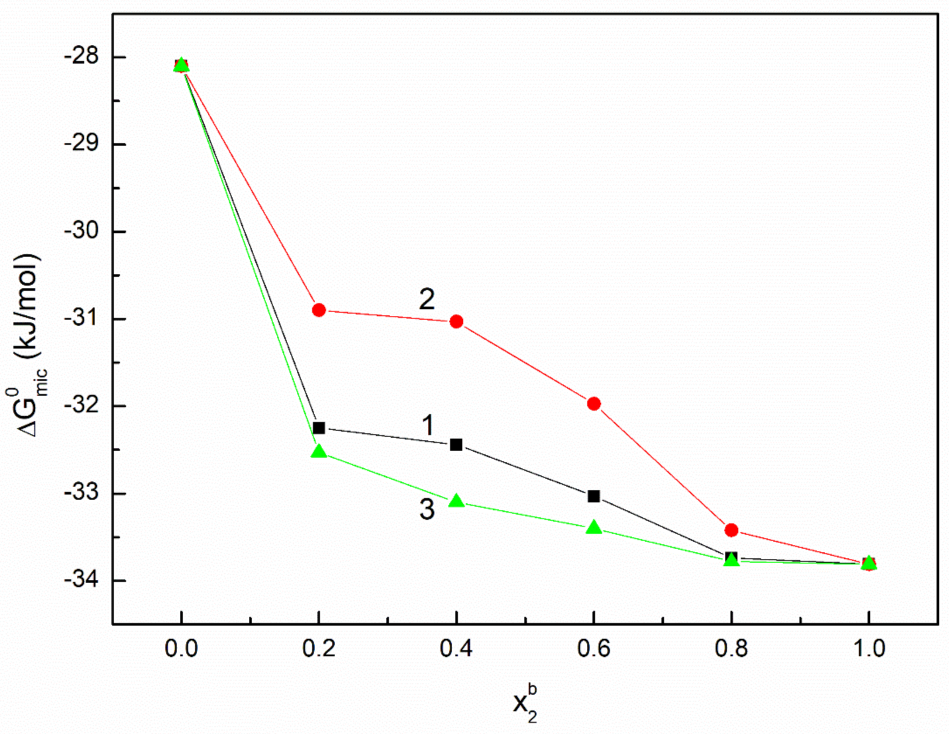

Figure S41. A plot of the Gibbs standard free energy of TX165 + RL micellization vs. the RL mole fraction in the mixture in the bulk phase (x_2^b) calculated from Eq. (21) (curve 1) and form Eq. (22) (curves 2 and 3). Curves 2 and 3 correspond to values calculated taking in the Eq. (22) x_1^b ,x_2^b and x_1^M, x_2^M, respectively.

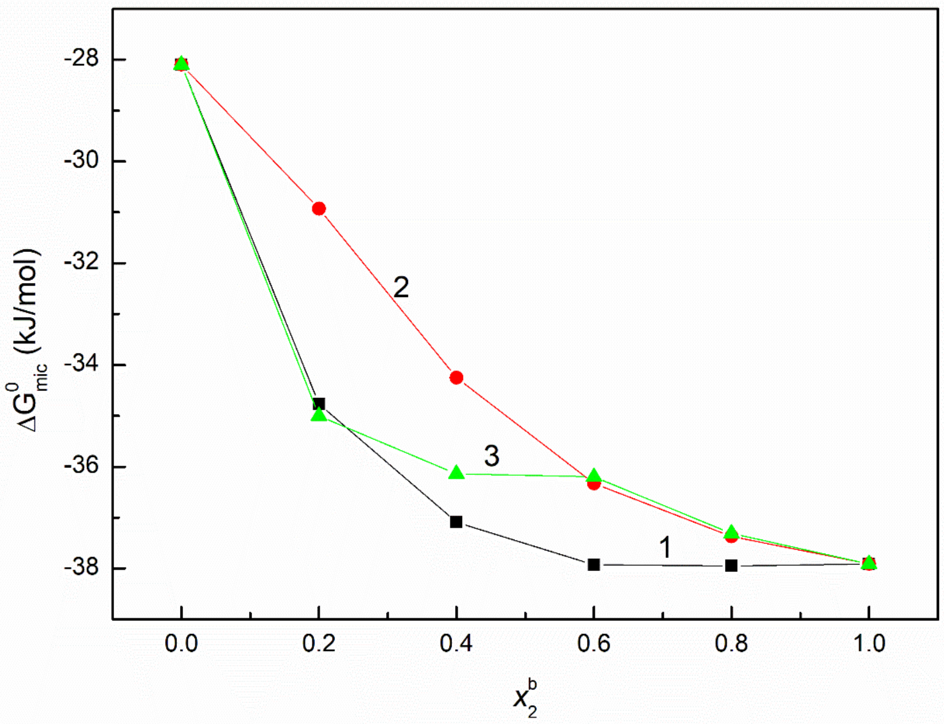

Figure S42. A plot of the Gibbs standard free energy of TX165 + SF micellization vs. the SF mole fraction in the mixture in the bulk phase (x_2^b) calculated from Eq. (21) (curve 1) and form Eq. (22) (curves 2 and 3). Curves 2 and 3 correspond to values calculated taking in the Eq. (22) x_1^b ,x_2^b and x_1^M, x_2^M, respectively.
